# Identification of biomarkers for pseudo and true progression of GBM based on radiogenomics study

**DOI:** 10.18632/oncotarget.10553

**Published:** 2016-07-13

**Authors:** Xiaohua Qian, Hua Tan, Jian Zhang, Keqin Liu, Tielin Yang, Maode Wang, Waldemar Debinskie, Weilin Zhao, Michael D. Chan, Xiaobo Zhou

**Affiliations:** ^1^ Department of Radiology, Wake Forest School of Medicine, Winston-Salem, NC 27157, USA; ^2^ School of Life Science, Xi'an Jiaotong University, Xi'an, Shanxi 710049, China; ^3^ The First Affiliated Hospital, Xi'an Jiaotong University, Xi'an, Shanxi 710061, China; ^4^ Department of Radiation Oncology, Wake Forest School of Medicine, Winston-Salem, NC 27157, USA

**Keywords:** GBM, pseudo and true tumor progression, radiogenomics, IRF9, XRCC1

## Abstract

The diagnosis for pseudoprogression (PsP) and true tumor progression (TTP) of GBM is a challenging task in clinical practices. The purpose of this study is to identify potential genetic biomarkers associated with PsP and TTP based on the clinical records, longitudinal imaging features, and genomics data. We are the first to introduce the radiogenomics approach to identify candidate genes for PsP and TTP of GBM. Specifically, a novel longitudinal sparse regression model was developed to construct the relationship between gene expression and imaging features. The imaging features were extracted from tumors along the longitudinal MRI and provided diagnostic information of PsP and TTP. The 33 candidate genes were selected based on their association with the imaging features, reflecting their relation with the development of PsP and TTP. We then conducted biological relevance analysis for 33 candidate genes to identify the potential biomarkers, i.e., Interferon regulatory factor (IRF9) and X-ray repair cross-complementing gene (XRCC1), which were involved in the cancer suppression and prevention, respectively. The IRF9 and XRCC1 were further independently validated in the TCGA data. Our results provided the first substantial evidence that IRF9 and XRCC1 can serve as the potential biomarkers for the development of PsP and TTP.

## INTRODUCTION

Gliomas are the most common primary brain tumors, representing about 30% of all brain and central nervous system tumors and 80% of all malignant brain tumors [1]. Among them, glioblastoma multiform (GBM) is classified as Grade IV/IV gliomas by the World Health Organization (WHO), accounting for approximately 50% of all glial tumors [2]. The incidence of GBM is approximately 3 cases per 100,000 person life-years in Europe and North America [3]. GBM is inherently aggressive tumor, with a median survival period of only 14–16 months and a 2-year survival rate of 26–33% [4], despite standard multimodal treatment of surgery and then concurrent radiotherapy and chemotherapy finally followed by adjuvant chemotherapy with the alkylating drug temozolomide (TMZ).

Although the intense chemotherapy with TMZ had clearly demonstrated a statistically significant survival benefit, this therapy increased occurrence of equivocal imaging findings, i.e., true tumor progression (TTP) versus treatment effect, which is referred to as pseudoprogression (PsP) [5]. PsP has been commonly defined as a subacute and post-treatment reaction with increasing contrast enhancement and vasogenic edema that mimics tumors progression at the tumor site or resection margins, but subsequently regresses or becomes stable without changes in treatment [6, 7]. This phenomenon occurs in roughly 20% of patients with recurrent GBM [5]. PsP is indistinguishable from early true tumor progression based on current imaging techniques. Although pathological confirmation of PsP is the gold standard, it is not a commonly appliable approach in clinical practice, as it requires a second surgery. Follow-up imaging provides the best indication of PsP [8] and is usually employed to make a diagnosis of PsP. But it takes several months to get a satisfactory diagnostic accuracy, which apparently impacts the clinical management of patients.

Over the last decade, PsP diagnosis has been recognized as a significant issue. A delayed decision makes the patients with PsP involved in the additional and unnecessary therapies. Similarly, an incorrect diagnosis of a TTP could result in erroneous termination of an effective treatment, with a potentially negative influence on patient's survival. To avoid these problems, novel, and reliable imaging techniques or diagnostic biomarkers are required for distinguishing PsP from TTP. Therefore, *the purpose of this study is to discover the potential genetic biomarkers and the biological mechanisms underlying PsP and TTP.* The potential genetic biomarkers, detected from GBM at initial surgery, can be employed to predict the development of the PsP and TTP for patients with GBM following standard post-treatment.

Several genetic and molecular markers involved in GBM have been found to associate with the development of PsP. Among these biomarkers, the MGMT promoter methylation obtains the most attention [5, 9–13], although its predictive value remains debatable [14–18]. Brandes et al. found that among 50 patients with early tumor progression, 21 (91%) of 23 patients with MGMT promoter methylation developed PsP, as compared to only 11 (41%) of 27 patients with unmethylated MGMT promoter [9]. Nevertheless, the sensitivity and specificity of MGMT promoter methylation status for detecting PsP were 66% and 89% respectively.

Other studies indicate that Ki67 expression [13], IDH1 mutation [14] and p53 mutation [19] are associated with the development of PsP and their clinical significance still remains to be confirmed [5]. Ki67, a marker of cellular proliferation, was differentially expressed in PsP and TTP groups [13]. The high level of cellular proliferation was associated with the development of PsP and emerged as a potential marker for distinguishing PsP from TTP. IDH1 mutation was regarded as a potential biomarker for discriminating between PsP and TTP in patients with GBM treated by standard therapeutics [14]. Its sensitivity and specificity were 66.7% and 100%. Additionally, Kan et al. observed that 7 (87.5%) out of 8 patients with PsP had overexpressed p53, which was only occurred in 3 (30%) out of 10 patients with TTP [19]. The authors postulated that overexpression of p53 was a potential biomarker for predicting the development of PsP. In contrast, Pouleau et al. concluded that the expression level of p53 had no predictive value for identification of PsP, although examined data were not presented [13].

**Figure 1:**
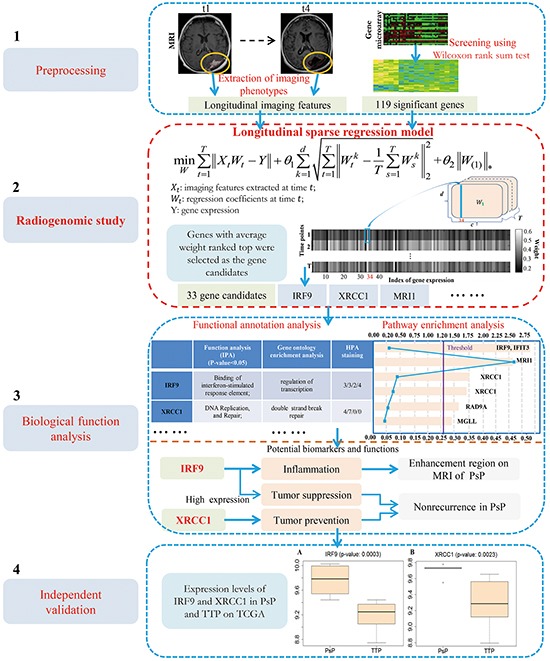
The proposed framework for biomarkers identification of PsP and TTP

To identify reliable biomarkers associated with the PsP and TTP, the clinical records, longitudinal MRI data, and genetic data (i.e., gene expression and methylation) were integrated in this study (Figure 1). To the best of our knowledge, this is the first time to introduce the radiogenomics research, i.e., the association between longitudinal imaging features and gene expression, in the selection of candidate genes for PsP and TTP. Specifically, a series of morphological features were extracted from the contrast-enhanced and necrotic regions on the contrast-enhanced T1MRI fluid-attenuated inversion recovery (FLAIR). The variance of morphological features along the longitudinal imaging can convey diagnostic information of PsP and TTP. Then, a novel longitudinal sparse regression model was developed to construct the relationship between imaging features and gene expression to select the candidate genes. Finally, biological function analysis was utilized to identify potential biomarkers, e.g., IRF9 and XRCC1. The potential biomarkers were further validated on an independent dataset from TCGA.

Overall, the major contribution of the proposed scheme can be summarized as follows. First, our private and public datasets, including clinic, radiology, and genomics data, provide a solid foundation for the comprehensive and reliable investigations. Second, we are the first to introduce the radiogenomics in the selection of candidate genes for differentiation of PsP and TTP. In the longitudinal regression model, we emphasized the consistent association between gene expression and imaging features at different time points. The clinical significance of our work can be summarized as (1) the identified two potential biomarkers, i.e., IRF9 and XRCC1, had significant expression difference between PsP and TTP groups, demonstrating the two genes can distinguish the PsP and TTP; and (2) the identified biomarkers were most associated with the longitudinal imaging features, which reflected the development of PsP and TTP, and their biological functions were mainly involved in the tumor suppression, tumor prevention, and inflammation. Thus, the biological mechanisms of potential biomarker IRF9 and XRCC1 were associated with the development of PsP and TTP.

## RESULTS

As shown in Figure 1, the study is designed with four phases, including i) extraction of imaging features and screening of differentially expressed genes; ii) radiogenomics study for selection of candidate genes; iii) biological function analysis for identification of potential biomarkers, and iv) validation for the identified biomarkers using an independent dataset.

### Imaging phenotypes extraction results

**Figure 2:**
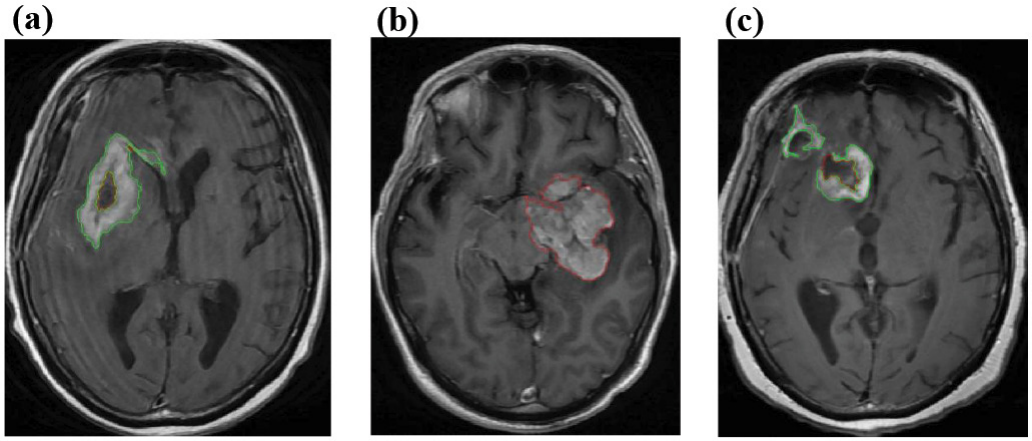
Segmentation of GBM. The outlined hyperintensity regions in **a.** and **c.** were enhanced regions and the outlined hypointensity regions in **a, b.** and **c.** were necrostic regions.

In clinical, the change of morphological features of tumor regions on follow-up MR imaging can serve as an indicator for the development of PsP and TTP. We extracted morphological features from the tumor regions on the Contrast enhanced T1 Flair MR Imaging as phenotypes for radiogenomic study. First, we outlined the enhancement (hyperintensity region) and necrotic regions (hypointensity region) using the proposed segmentation scheme (Materials and Methods), as shown in the Figure 2. Then, we extracted 225 morphological features from segmented tumor regions, such as the major/minor axis length of enhanced region and the thickness of enhancing margin. These features, shown in Supplementary Table S1, were potentially useful in discriminating PsP and TTP.

### Genes significantly related to PsP and TTP

**Figure 3:**
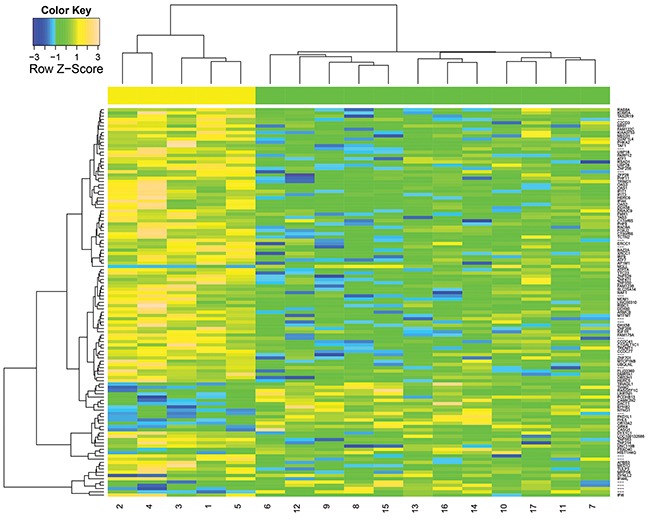
Hierarchical clustering of 119 differentially expressed genes (*P*<0.005) between PsP and TTP groups The IDs numbered 1 to 5 represents the data from PsP cases and 6-17 denotes the TTP cases.

We initially identified 119 genes with *p*<0.005 using the Wilcoxon rank sum test from totally 22011 genes, as shown in Supplementary Table S2. To illustrate the difference between PsP and TTP, we then performed hierarchical clustering analysis for the identified genes. As shown in the Figure 3, the numbers 1 to 5 display the results of tumor sampled collected from patients with PsP and 6 to 17 for those with TTP. Obviously, 119 genes were differentially expressed in two groups, indicating that these genes can be used as candidates for categorizing samples with early tumor progression into PsP or TTP. It is also easy to observe the hypo- or hyper-expression of significant genes in PsP or TTP groups.

### Selection of candidates genes from radiogenomics analysis

**Figure 4:**
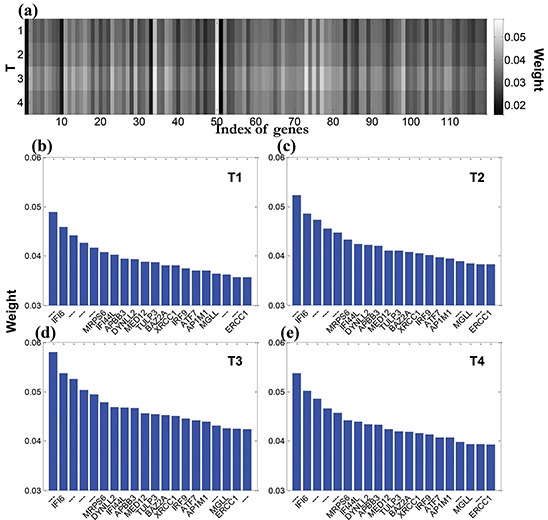
Illustration of the association between the imaging features and differentially expressed genes **a.** overall weight map of 119 genes with respect to 225 imaging features; **b-e.** show the genes with top-ranked weights (20) across the longitudinal imaging time T1-T4.

The 119 differentially expressed genes screened by the Wilcoxon random test were then used for candidate gene selection. We used the multi-task longitudinal sparse regression method to reveal the associations between the 119 genes and 225 morphological features extracted from the longitudinal MRI. The trade-off parameters θ_1_ and θ_1_ in the regression model (Eq. (6) in Materials and Methods) were set from 0.1 to 50. We obtained the overall weight map of 119 genes with respect to 225 imaging features, as shown in the Figure 4(a). The genes with big weights have distinct patterns that span across all the four-time points, which shows the influence of these genes are longitudinally stable. Figure 4(b)-(e) show the genes with top-ranked weight across the longitudinal imaging time. Obviously, the genes, such as IFI6, IRF9, XRCC1, presented at all four-time points, although their weights were slightly different from time to time.

To ensure the identified candidate genes insensitive to the parameters in our longitudinal sparse regression model, we first set the trade-off parameters θ_1_ = θ_2_ with a serial range [0.1 0.2 0.5 1 2 5 10 20 50], and the number of iteration was 800, 1000, or 1200. We then calculated the average weights for each gene over the four-time points. The genes with weights ranked top 50 were selected with each combination of parameters. We defined a coverage rate *P* as the occurrence frequency of genes on the lists of weights ranked top 50 in 27 parameter combinations. The genes selected for more than 22 times with a coverage ratio *P* >80% were considered as the candidate genes for post-biological analysis and listed in Table 1.

**Table 1:** Candidate genes identified by the coverage rate *P*, which means the occurrence frequency of genes on the lists of weight ranker top 50 in 27 parameter combinations

	Identified candidate genes	Total
P=0.8 (>22)	AP1M1; C19orf66; IFI44L; IFIT3; IRF9; OAS3; TULP3; USP18; DYNLL2; TAS2R19; APBB3; FAM122C; IFI6; MED12; MGLL; BAZ2A; ERCC1; PHF8; RAD9A; XRCC1; ATF7; C17orf65; C2CD3; DNAJC9; FMR1; FOXJ2; KDM5A; KIAA0753; MRI1; MRPS6; RAB8A; TAB3; TCTN2	33
P=0.9 (>24)	AP1M1; C19orf66; IFI44L; IRF9; TULP3; DYNLL2; APBB3; IFI6; MED12; BAZ2A; ERCC1; PHF8; XRCC1; ATF7; C2CD3; DNAJC9; MRPS6; RAB8A; TAB3; TCTN2	20
P=1 (==27)	AP1M1; C19orf66; IFI44L; IRF9; TULP3; DYNLL2; APBB3; IFI6; XRCC1; ATF7; DNAJC9; MRPS6	12

### Biological relevance and characterization of candidate genes

We then performed biological relevance analysis for the candidate genes to identify potential biomarkers for the PsP and TTP cases. The biological function analysis consisted of i) functional annotation and pathway enrichment analysis, and ii) identification of potential biomarkers.

#### Result of functional annotation and pathway enrichment analysis

**Figure 5:**
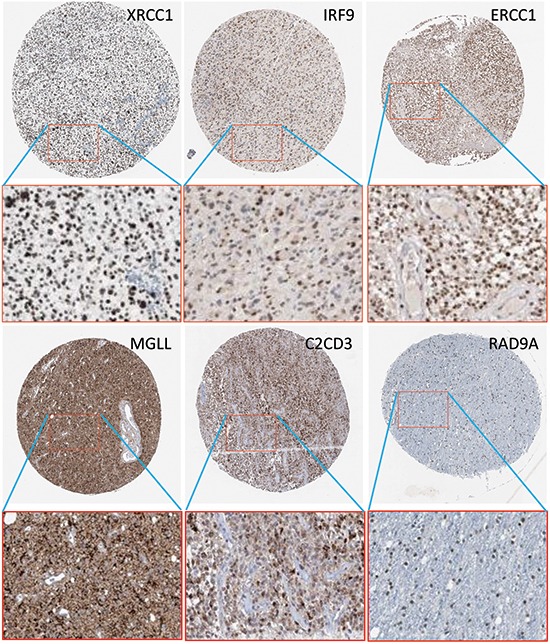
Protein expressions of IRF9, XRCC1, ERCCC1, MGLL, C2CD3, and RAD9A in corresponding antibody-stained images from Human Protein Atlas.

The functional annotation analysis results for the representative candidate genes is shown in Table 2. IPA and GO were used to explore the main biological annotations of candidate genes associated with cancer development. The functional annotations from IPA were filtered by a p-value<0.05 using the fish exact test. The functional annotations from IPA (second column in Table 2) and GO (third column in Table 2) databases indicated that most of these genes have roles in cell cycle, death, and survival, etc. and are directly or indirectly related to cancer development. For example, IRF9, transcription factor, participates in the signaling transduction pathway of type 1 interferon, serving as a cancer suppressor. Besides, the fourth column describes the protein levels of representative genes in GBM sections from HPA. The protein levels of IRF9, XRCC1, MGLL, ERCC1, C2CD3, RAD9A, and MRI1 can be found high in the GBM tissues, whereas USP18 only exists in the normal tissue. Figure 5 shows the protein expression of six representative genes in corresponding antibody-stained images from HPA.

**Table 2:** Functional annotation analysis using IPA, GO, and HPA databases

Function analysis (IPA) (Fish Exact test P-value<0.05)		Gene ontology enrichment analysis	HPA staining (High/medium/ low/none)
IRF9	Binding of interferon-stimulated response element; Expression of RNA	Type I interferon biosynthetic process; regulatory region DNA binding; regulation of transcription, DNA-templated cytoplasm	3/3/2/4
XRCC1	DNA Replication, Recombination, and Repair; Cell cycle, Cell Death and Survival	Negative regulation of mitochondrial DNA replication; Single/double strand break repair	4/7/0/0
MRI1	N/A	L-methionine biosynthetic process from methylthioadenosine;	2/1/4/5
MED12	Initiation of expression of RNA; development of central nervous system	Transcription factor binding; positive regulation of transcription from RNA ; polymerase II promoter	0/3/3/5
MGLL	Cellular Movement: migration of pancreatic cancer cells; Cell-To-Cell Signaling and Interaction, Nervous System Development and Function	Regulation of signal transduction; regulation of endocannabinoid signaling pathway	1/2/2/7
ERCC1	DNA Replication, Recombination, and Repair; Cell Morphology, Cellular Function and Maintenance;	Damaged DNA binding; replicative cell aging; cell development; protein binding	1/9/0/1
RAD9A	Cell Cycle, DNA Replication, Recombination, and Repair; expression of RNA; Cell Death and Survival, Cellular Growth and Proliferation, Embryonic Development	DNA repair; protein binding; positive regulation of intrinsic apoptotic; signaling pathway in response to DNA damage; DNA replication	2/7/1/0
C2CD3	Development of central nervous system; Cancer, Organismal Injury and Abnormalities, Reproductive System Disease	Protein binding; regulation of smoothened; signaling pathway regulation of proteolysis	1/8/1/2
ATF7	Nervous System Development and Function; Cell Death and Survival; expression of RNA	Negative regulation of transcription from RNA polymerase II promoter; DNA binding transcription factor activity involved in negative regulation of transcription	0/1/0/10
KDM5A	Cellular Development, Cellular Growth and Proliferation; Cancer, Organismal Injury and Abnormalities; Reproductive System Disease: metastasis of adenocarcinoma cell lines	DNA binding; positive regulation of transcription, DNA-templated; chromatin binding; zinc ion binding	0/5/6/1
USP18	replication of RNA; Cellular Growth and Proliferation, Tissue Development	ISG15-specific protease activity; regulation of type I interferon-mediated signaling pathway;	0/0/0/11
TULP3	Development of central nervous system	Protein localization to photoreceptor outer segment; brain development	0/0/1/11
IFIT3	Cancer, Organismal Injury and Abnormalities, Reproductive System Disease	Negative regulation of apoptotic process; negative regulation of cell proliferation; type I interferon signaling pathway	0/8/4/0

**Figure 6:**
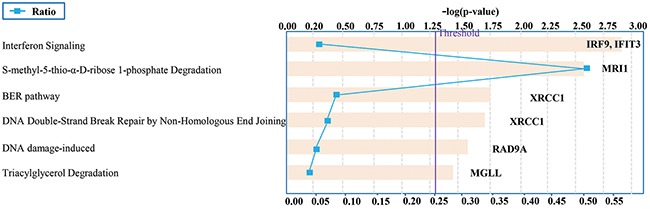
Top-ranked canonical pathways associated with the candidate genes selected by radiogenomics Canonical pathways are ordered by the p-values (p<0.05).

IPA was also applied for pathway analysis of 33 candidate genes. The impact-value threshold calculated from pathway topology analysis was set to 0.05, and the generated pathways above this threshold were filtered out as the significant ones. The generated canonical pathways are presented by the order of significance in Figure 6. Three out of the six most significant canonical pathways were related to the cancer suppression or prevention, namely Interferon Signaling (IRF9, IFIT3), BER pathway (XRCC1) and DNA Double-Strand Break Repair by Non-Homologous End Joining (XRCC1).

#### Potential biomarkers for PsP and TTP

These candidate genes were directly or indirectly related to cancer development. Among them, IRF9 and XRCC1 attracted our attention and were identified as the potential gene biomarkers for PsP and TTP, based on the results from the functional annotation, pathway enrichment analysis, and published data. We further characterized the functions of IRF9 and XRCC1 in tumor development.

IRF9, also known as Interferon-stimulated gene (ISGF3γ) or p48, belongs to the IRF family [20]. It participates in a series of biological pathways related to cell proliferation, apoptosis, and innate immunity [20–26]. The role of IRF9 in tumor suppression is mainly implicated in the context of type I Interferon (IFN)-mediated antitumor activities through Janus Kinases/STAT pathway [23], as shown in Supplementary Figure S1. More specifically, IFN-α, a member of type I IFN, has been clinically used in the treatment of certain malignancies, such as malignant melanoma and renal cell carcinoma. IFN-α signals through the JAK-STAT pathway and initiates transcription of a group of genes, such as tumor necrosis factor-related apoptosis-inducing ligand (Trail), which induces apoptosis. The transcription induced by IFN-α predominantly required IRF9. Among the intracellular JAK-STAT pathway components, IRF9 is a crucial regulator in eliciting the antiproliferative activity of IFN-α [25]. For example, IRF9 overexpression facilitated IFN-α induced apoptosis in T98G (human GBM) cells [25].

XRCC1, one of the DNA repair genes, is a critical factor in the base excision repair (BER) pathway, which is one of major DNA maintenance mechanisms [27]. XRCC1 encodes a scaffold protein and functions as a coordinator in BER pathway by forming a complex between DNA polymerase beta, DNA ligase III and poly (ADP-ribose) polymerase [28]. DNA repair capacity plays a critical role in maintaining genome integrity and preventing carcinogenesis [28, 29]. XRCC1 deficiency results in increasing frequencies of gene mutation and chromosomal aberrations, in turn increasing the risk of cancer [30, 31]. In contrast, increased expression of XRCC1 is a benefit to the repair of DNA damages by chemicals or radiation, thereby maintaining genomic stability and integrity[32]. A recent meta-analysis suggested XRCC1 variations were associated with increased risk of GBM [33].

**Figure 7:**
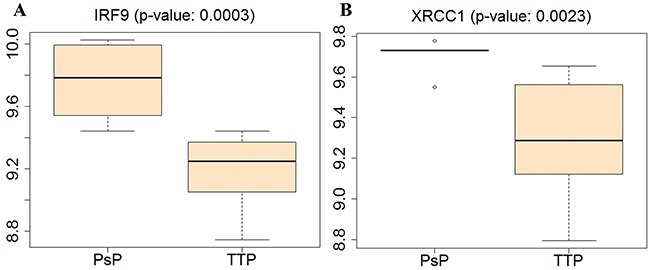
Boxplot of gene expressions for IRF9 and XRCC1 in PsP and TTP groups

Figure 7 shows that the expression levels of IRF9 and XRCC1 in the PsP group were higher than those in TTP group. The high expression of IRF9 can elicit the antiproliferative activity of IFN- α with the induction of apoptosis in GBM cells [25], and suppress the tumor progression. Similarly, XRCC1 with high expression can maintain genomic stability and integrity, and prevent cancer. The invasive GBM cells in the resection margin or within 2 cm of the resection cavity shows a decrease in their rate of proliferation and a relative resistance to apoptosis, thus higher expression levels of IRF9 and XRCC1 in PsP group may contribute to the non-recurrence of GBM. Moreover, IRF9 overexpression is associated with the inflammation recognized as the PsP of tumor [34, 35]. These phenomena were consistent with our hypothesis that genomic profiles are associated with the recurrence of GBM after standard treatment (i.e., development of PsP and TTP). Therefore, it is tenable to consider the IRF9 and XRCC1 as the potential biomarkers for PsP and TTP.

### Potential biomarkers’ expression in TCGA

**Figure 8:**
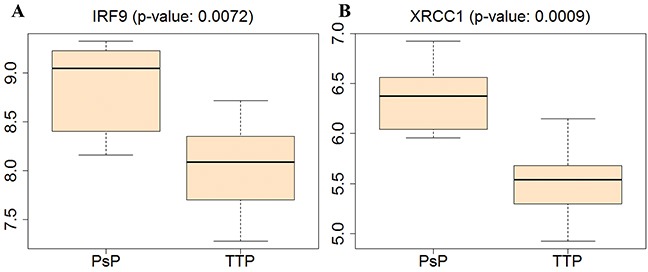
Validations for expression levels of IRF9 and XRCC1 in PsP and TTP groups on independent dataset, i.e., 6 cases of PsP and 15 cases of TTP, from TCGA

Our analysis indicated that the the higher expression levels of IRF9 and XRCC1 were tightly associated with PsP cases. To further validate our finding, we collected another independent TCGA data, including 6 PsP samples and 15 TTP samples and analyzed the expression levels of above genes. As shown in the Figure 8, the expression level of IRF9 and XRCC1 are significantly higher in the PsP group than those in the TTP, consistent with the results obtained usingthe dataset from our medical center.

## DISCUSSION

In the present study, we integrated the clinical, radiological, and genomic data for identifying the potential genetic biomarkers associated with the development of PsP and TTP. First, the differentially expressed genes were initially filtered by Wilcoxon rank sum test; second, the 33 candidate genes were selected by radiogenomics analysis; and third, the potential genetic biomarkers were further identified and analyzed based on their biological function. Specifically, IRF9 and XRCC1 were highly expressed in the tumors from PsP patients and both of them are involved in cancer suppression and prevention. Therefore, IRF9 and XRCC1 were finally identified as the potential biomarkers for PsP and TTP. The relative expression levels of IRF9 and XRCC1 were also successfully validated in another independent data set from TCGA.

Radiogenomics research mainly refers to the relationship between the patient genetics and imaging characteristics [36]. Exploring this relationship will be useful for understanding the development of cancer and tissue response to the treatment. For examples, the association map from the radiogenomics can be used to decode gene expression [37], build the prognostic imaging signatures [38], and identify the specific genetics impacting the radiologic features [39]. In this study, we are the first time to introduce the radiogenomics analysis, i.e., association study between cancer imaging features and gene expression using our developed sparse longitudinal regression model, for selection of candidate genes for PsP and TTP. Clinically, the change of imaging features regarding the contrast-enhanced region and necrosis along the longitudinal MRI can be used to diagnose the PsP and TTP. Thus, 33 candidate genes closely associated with the imaging features from PsP and TTP could reflect the development of PsP and TTP. This is our main methodological contribution of this study. Moreover, the selected candidate genes from radiogenomics were insensitive to the parameters in the sparse longitudinal regression model. Trade-off parameters θ_1_ and θ_2_ were set equally with a selectable range [0.1 0.2 0.5 1 2 5 10 20 50], and the number of iteration was 800, 1000, or 1200. In these 27 types of parameter combination, the genes with weight ranked top 50 for more than 22 times were selected as the candidate genes. In fact, the finally identified potential biomarker genes, i.e., IRF9 and XRCC1, have high weight gains, as shown in Table 1. In addition, we have conducted the comparison with classic scheme (i.e. directly using the sample labels for marker identification) in the Supplementary Materials. In summary, the radiogenomics study based on the differentially expressed genes facilitates the biological analysis in efficiently identifying most relevant signaling pathways, as shown in Supplementary Figure S2 and S3.

We explored the classification performance of differentially expressed genes and the effect of morphological features with different sizes. Figure 3 shows these 119 differentially expressed genes were differentially expressed in two groups, indicating these genes can be used as candidates for categorizing samples into PsP or TTP. Thus, We investigated the classification ability of 119 differentially expressed genes using Support Vector Machine (SVM) algorithm [40] with fivefold cross-validation. We obtain the 100% accuracy with area under receiver operating characteristic curve (AUC)=1, as shown in Supplementary Figure S4. We also investigated the effect of morphological features with different sizes in the Supplementary Materials. Figure S5 and Supplementary Table S3 show a subset of these morphological features (i.e., 20 or 50 features used in clinical practices) may identify the same set of biomarkers with optimal parameters for our longitudinal regression model. However, the results of our model with a subset of these morphological features were dependent on the parameters, while 255 features produced the robust performance, which is insensitive to the parameters.

The potential limitation of this study is the sample size. The dataset used in this study includes clinic records, longitudinal MRI, and genomic data (i.e., gene expression and methylation) from individual patients. It is difficult to simultanously collect all types of the data. However, the validation using the datasets from other sources (i.e., TCGA and TCIA) confirmed our findings.

IRF9 plays a vital role in suppressing tumor in multiple ways. IRF9 can act as a critical component of IFN-induced p53 upregulation process, which contributes to boosting the activation of the p53 mediated proapoptotic pathway upon stimulation with DNA-damaging agents such as radiation and chemotherapeutic agents [21]. IRF9 also can work as a regulator [23, 24] without dependent on IFN. It directly binds to the promoter region of Sirt1 to inhibit its expression, which enhances the activation of P53 to exacerbate cell death [22]. Interestingly, overexpression of IRF9 in half of the breast and uterine cancer tumor was observed, indicating that IRF9 may be important in signaling transductions in these tumor types [26]. These studies further corroborated the potential role of IRF9 in cancer biology.

Methylation in MGMT promoter region has widely been suggested as a biomarker associated with the development of PsP in previous studies [5, 9–13]. Brandes et al. claimed that MGMT promoter status could be used to predict PsP in methylated cases with 91% accuracy [9]. To date, the results from other studies were not consistent with Brandes's conclusion [14–18], as shown in the Supplementary Table S4. The prediction accuracies of PsP in the methylated cases were 91%, 40%, 37.5%, 37.5% and 80%, respectively. Pinho et al. found no correlation between MGMT promoter's methylation and development of PsP, although they did not show the prediction accuracy [16]. These studies indicated that taking MGMT promoter's methylation as the biomarker for PsP is still controversial. The limited availability of samples may lead to inconsistent conclusions. The sample numbers in Supplementary Table S4 were 50, 11, 73, 55, 25, respectively, which brought out the bias results. Another possible reason for the inconsistent results is the so-called mutual exclusivity among a set of genes which take effect in same or similar biological processes, for instance, in one signaling pathway [41, 42]. The mutual exclusivity principle claims that, typically, only one gene in a functionally correlated gene set will exemplify abnormality in a disease, depending on the cell types and physiological conditions of specific contexts. MGMT is one of several DNA repair genes reported in the existing literatures. Therefore, it is not surprising that different research group reported different results about DNA repair gene abnormality. In addition, a failed Phase II clinical trial indicated that GBM patients may not benefit from the MGMT silence merely. MGMT did not show significant restoration of TMZ sensitivity in patients with TMZ-resistant GBM [43].

Both MGMT and XRCC1 are involved in DNA repairs and exert very similar effect on relevant cells [44], it is possible that only one of these two genes was identified as biomarker for PsP and TTP in particular studies according to the mutual exclusivity theory. In terms of gene methylation and promoter methylation status of MGMT, XRCC1, and IRF9, there was no significant difference in the two groups in our data, as shown in Supplementary Figure S6. Considering the fact that previous studies reported very inconsistent results about DNA methylation for the well-known TTP-related MGMT gene, we postulate that the progression of PsP and TTP is irrelevant to DNA methylation, and the difference in gene expression is not caused by methylation variation, but other factors to be elucidated.

Similar to MGMT, XRCC1 is also a DNA repair gene. Previous studies based on the data collected post- chemotherapy revealed that high expression of DNA repair gene resulted in resistance to TMZ treatment [45], which did not contradict with our conclusion that XRCC1 with high expression can decrease the risk of cancer. In fact, XRCC1 plays a dual role at different treatment phases of GBM. XRCC1 with high expression can not only prevent cancer prior to the radiation therapy but also promote the resistance to TMZ. The XRCC1 expression can be deemed as a response to the treatment of radiation and chemotherapy, which is a dynamic process. In this study, we employed the genomic data collected in the surgery sample prior to radiotherapy and/or chemotherapy to identify the causative genes for prediction of PsP and TTP, while the data of XRCC1 expression after chemotherapy was unavailable. Overall, the essential function of XRCC1 is DNA repair, and its dual role depends on different treatment stages.

In summary, we introduced a longitudinal sparse regression model to construct the relationship between imaging features and gene expressions for selection of candidate genes, among which the potential biomarkers, i.e., IRF9 and XRCC1, were further identified for PsP and TTP. IRF9 and XRCC1 can be employed as a predictor to assist classifying the PsP and TTP since they were significantly differentially expressed in PsP and TTP. Additionally, the biological mechanisms of IRF9 and XRCC1 underlying tumor suppression, prevention, and inflammation were carefully addressed. Collectively, IRF9 and XRCC1 as potential genetic biomarkers were closely associated with the development of PsP and TTP.

## MATERIALS AND METHODS

### Data collection

This study was approved by the Institutional Review Board of Wake Forest School of Medicine. We totally collect 38 patient samples, among which 17 samples were from our hospital, and 21 samples were from Cancer Imaging Archive (TCIA) [46] and the Cancer Genome Atlas (TCGA, http://cancergenome.nih.gov/). The private data (17 samples) were used for biomarker identification, and the public data (21 samples) were applied for biomarker validation.

#### Private data for biomarkers identification

The clinical records, longitudinal imaging data, and biological datasets (i.e., gene expression, and methylation) for GBM patients were collected at Wake Forest School of Medicine. A total of 17 patient samples (including 5 PsP and 12 TTP patients) with all of the four types of data were selected for our analysis. These patients carried histologically proved GBM (World Health Organization classification, grade IV) and presented apparent early tumor progression according to the conventional MR imaging following the standard postsurgical treatment of radiation and chemotherapy with temozolomide (TMZ). All of the enrolled patients (June-2007 to February-2010) received a similar dose (around 60 Gy) of conformal radiation therapy (CRT). The collected patient data also included information regarding age, sex, date of surgery, radiotherapy (RT) starting date, RT completion date, date of failure or progression, pseudo-progression, and date of death, etc.

Longitudinal imaging data were collected by magnetic resonance scanners (GE Medical System, repetition time: 17204-27431 ms; echo time: 7.0-26.2 ms; 2-5-mm slice thickness; 0.42-0.46-mm in plane resolution; and 512*512 matrix). Four contrast-enhanced T1MRI FLAIR for each patient were acquired from the initiation of CRT to the diagnosis date of PsP or tumor progression.

The gene expression levels of GBM patient were measured by Affymetrix Human Exon 1.0 ST (HuEx-1_0-st-v2) arrays. All the probe sets’ locations were obtained from the annotation file which was downloaded from Affymetrix website. The DNA methylation was determined using the Illumina Infinium humanMethylation 450k BeadChips and quantified according to the beta values. The beta values were ranged from 0 to 1. A β value close to 1 was considered a high level of DNA methylation.

#### Public data for biomarkers validation

We also collected 21 clinical samples with longitudinal images and corresponding gene expression data from TCIA and TCGA, respectively. The TCIA contains imaging data for a subset of patients from TCGA. The same patient has a unique sample ID in the two databases. First, a medical physicist categorized the TCIA samples into PsP and TTP groups based on the longitudinal MRI, according to the definition of PsP, i.e., the contrast enhancement regresses or becomes stable on longitudinal MRI, as shown in Supplementary Figure S7. As a result, we obtained 6 PsP and 15 TTP samples, and the sample IDs were presented in the Supplementary Table S5. Then, we collected the gene expression data of the corresponding samples from TCGA.

### Extraction of imaging phenotypes

The quantitative morphological features from tumors along longitudinal MRIs could be used for evaluating PsP and TTP [8]. We extracted morphological features from the tumor regions on the Contrast enhanced T1 Flair MR Imaging. This process consisted of semi-automatic segmentation of tumor regions and extraction of morphological features.

The semi-automatic tumor segmentation can be decomposed into four steps, including alignment, segmentation, identification and refinement, as shown in Supplementary Figure S8. First, the inclination angle and position of the head region were corrected by aligning the midsagittal line with the vertical centerline of the images [47, 48]. We applied a two-step scheme to segment the enhanced (hyperintensity region) and necrotic regions (hypointensity region) using a level set method [49]. A user interaction, i.e., only one mouse click, is required to locate the enhanced regions or necrosis regions in the segmented regions for each case [50]. The largest connected region was then identified as the enhanced or necrotic regions. To provide the reliable and accurate results for features extraction, we then manually refined the unsatisfied segmentation.

Once obtained the enhanced and necrotic regions, we extracted a series of morphological features from them. We calculated area, thickness, length, radius, regions number, and area proportion of enhanced region and necrotic region for each slice. The enhanced or necrotic region with the largest area was selected as the primary region, whose area, perimeter, area of the bounding box, major axis length, minor axis length, orientation, solidity, eccentricity, compactness, and sphericity were calculated [47]. Finally, we took features from the slice with the largest tumor area in a case, and calculated the mean, maximum, minimum, and sum of features from all the slices in a case as the morphological features.

### Screening of significantly differentially expressed genes

To obtain the differentially expressed genes, We compared the gene expression levels in the PsP and TTP groups. A Wilcoxon rank sum test was applied to determine if a gene was differentially expressed in two groups with a p-value less than 0.005. We then conducted the supervised hierarchical clustering and heatmap visualization of screened significant genes using R package. Briefly, significant genes were hierarchically clustered by average linkage using Pearson correlation as the distance metric and then visualized using default color saturation [51].

### Selection of candidate genes by radiogenomics study

We proposed a multi-task longitudinal sparse regression method to reveal the associations between imaging features extracted from the longitudinal MRI and gene expression profiles. A set of candidate genes closely correlated with the morphological features were demonstrated.

#### Multi-task longitudinal sparse regression model

Let $X=(X_{1},X_{2},\cdots ,X_{T})\in {ℜ}^{n\times d\times T}$ be the input longitudinal imaging features of the lesion area of GBM, which are extracted at *T* consecutive time points. *X_t_* is the imaging features extracted at time *t* (1 ≤ *t* ≤ *T*). Accordingly, *X* is a tensor data with *n* samples, *d* image features and *T* time points. Let $Y={\left(y_{1},y_{2},\cdots ,y_{n}\right)}^{T}\in {ℜ}^{n\times c}$ be the output genetic variations described by *c* gene expression values for the *n* samples, where the $y_{i}\in {ℜ}^{c}$ is the expression values of the *i*-th sample.

**Figure 9:**
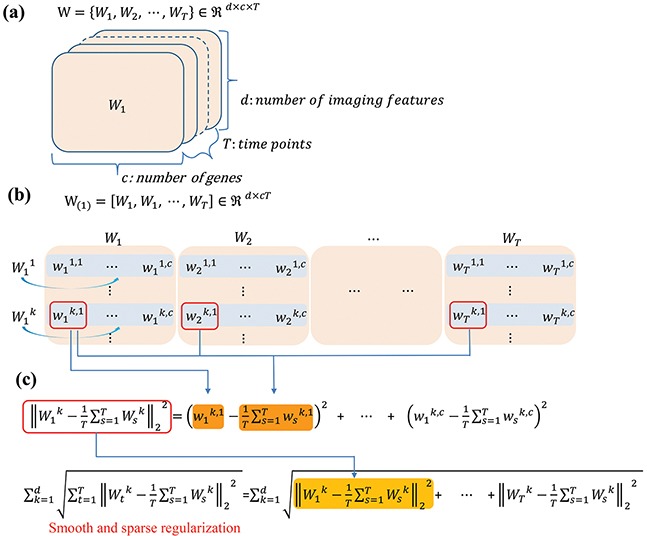
Schematic illustration of the regression coefficient matrix and regularization in longitudinal sparse regression model **a.** visualization of the regression coefficient *W* learned from the association study on longitudinal data; **b.** the coefficient matrix unfolded from W along the feature dimension; and **c.** the composition of smooth and sparse regularization.

A simple multivariate regression model can be used for association the imaging features with gene expression profile, which minimizes the following objective function:

$$\underset{W}{\min}\sum_{t=1}^{T}\left\|X_{t}W_{t}-Y\right\|+\theta \sum_{t=1}^{T}\sum_{k=1}^{d}{\left\|W_{t}^{k}\right\|}_{2}^{2}$$ (1)

where the first term measures the longitudinal loss, θ is the trade-off parameter. *W_t_^k^* denotes the *k*-th row of regression coefficients matrix *W_t_* at time *t*, as shown in the Figure 9(a) and (b), and measures the relative importance of the *k*-th feature at time *t* for predicting the response of the gene expression.

However, there are potential limitations of the regression model in Eq. (1) when applied for assessing the association imaging features with genetic changes as follows.

1. Tasks at different time points are assumed to be independent of each other; thus, this model cannot fully catch the relationship among tasks at different time points.
2. In the course of disease progression, it is reasonable to assume that the difference in the regression coefficients between successive time points is relatively small, whereas this model may yield fluctuated regression coefficients at successive time points for a patient.

Inspired by previous studies [52, 53], we first introduce a smooth and structured sparse regularization term into the longitudinal regression model to address these problems:

$$\underset{W}{\min}\overset{T}{\underset{t=1}{\sum }}\|X_{t}W_{t}-Y\|+\theta_{1}\overset{d}{\underset{k=1}{\sum }}\sqrt{\overset{T}{\underset{t=1}{\sum }}\|W_{t}^{k}-\frac{1}{T}\overset{T}{\underset{s=1}{\sum }}W_{s}^{k}{\|}_{2}^{2}}$$ (2)

where $\sum_{t=1}^{T}{\left\|w_{t}^{k}-\frac{1}{T}\sum_{s=1}^{T}w_{s}^{k}\right\|}_{2}^{2}$ in the second term of Eq. (2) is used to penalize the large deviations between regression coefficients and their means at different time points, in order to smooth the regression coefficient curve, as shown in Figure 9(c). The second term is essentially a group ℓ_2,1_ norm. The group ℓ_2,1_ norm can simultaneously perform regression analysis for the data from different time points. As a result, each type of imaging features with common influences to all of the genes across all the time points. The ℓ_1_-*norm* in the group ℓ_2, 1_-*norm* are used for the penalties crossing all of the groups to enforce the sparsity among groups. Therefore, only a small amount of image features are associated with a particular gene.

We then introduced the low rank norm to further reduce the redundant information since different imaging features are interrelated to each other and their effects during the association process could be overlapped [52]. Let $W_{\left(1\right)}=[W_{1},W_{2},\cdots ,W_{T}]\in {ℜ}^{d\times (c\times T)}$ as illustrated in the Figure 9(b), the rank of the coefficient matrix should be low. The trace norm is the best convex approximation of the rank norm [54]. Thus, we introduce the trace norm into the regression model as following:

$$\underset{W}{\min}\sum_{t=1}^{T}\left\|X_{t}W_{t}-Y\right\|+\theta_{1}\sum_{k=1}^{d}\sqrt{\sum_{k=1}^{d}{\left\|W_{t}^{k}-\frac{1}{T}\sum_{k=1}^{T}W_{t}^{k}\right\|}_{2}^{2}}+\theta_{2}{\left\|W_{\left(1\right)}\right\|}_{*}$$ (3)

where $\|W_{\left(1\right)}{\|}_{*}$ denotes the trace norm of a matrix and can be defined as

$$\|W_{\left(1\right)}{\|}_{*}=\overset{\min(n,c\times T)}{\underset{i=1}{\sum^{\;}}}\sigma_{i}(W_{\left(1\right)})=Tr{\left(W_{\left(1\right)}W_{\left(1\right)}^{T}\right)}^{1/2}$$ (4)

where σ_i_(*W*_(1)_) denotes the *i*-th singular value of *W_(1)_*.

The Eq. (4) is our proposed longitudinal sparse regression model. The optimization problem in Eq. (4) admits an analytical solution [52, 55]. Taking the derivative with respective to *W_t_*, and setting the derivative to zeros, we have

$$X_{t}^{T}X_{t}W_{t}-X_{t}^{T}Y+\theta_{1}(DW_{t}-D\bar{D})+\theta_{2}\bar{\bar{D}}W_{t}=0$$ (5)

where *D* is a diagonal matrix with the *k*-th diagonal element as $1/\sqrt{\sum_{t=1}^{T}\left||W_{t}^{k}|{|}_{2}^{2}-\frac{1}{T}|\right|\sum_{t=1}^{T}W_{t}^{k}|{|}_{2}^{2}}$, $\bar{D}=\frac{1}{T}\sum_{t=1}^{T}W_{t}$ and $\bar{D}=\frac{1}{2}{\left(W_{\left(1\right)}W_{\left(1\right)}^{T}\right)}^{-\frac{1}{2}}$. Thus, we have

$$W_{t}={\left(X_{t}^{T}X_{t}+\theta_{1}D+\theta_{2}\bar{\bar{D}}\right)}^{-1}(X_{t}^{T}Y+\theta_{1}D\bar{D})$$ (6)

We can calculate *W_t_*(1 ≤ *t* ≤ *T*) by Eq. (6), when the time t changes from 1 to *T*. The *D*, $\bar{D}$ and $\bar{\bar{D}}$ in the Eq. (5) are dependent on *W_t_*; thus, they are unknown variables. We proposed an iterative algorithm to solve this problem, as described in Algorithm 1.

Algorithm 1 **an iterative algorithm to solve the optimum problem in Eq. (6)**

**Input:** $X\in {ℜ}^{n\times d\times T}$.

**Output:** $W\in {ℜ}^{d\times c\times T}$

Initialize $W^{\left(0\right)}\in {ℜ}^{d\times c\times T}$

**While** not *converge* do

1. Calculate the diagonal matrix *D*, where the *k*-th diagonal is $1/\sqrt{\sum_{t=1}^{T}{\left\|W_{t}^{k}\right\|}_{2}^{2}-\frac{1}{T}{\left\|\sum_{t=1}^{T}W_{t}^{k}\right\|}_{2}^{2}}$
2. Calculate the $\bar{D}=\frac{1}{T}\sum_{t=1}^{T}W_{t}$
3. Calculate the $\bar{\bar{D}}=\frac{1}{2}{\left(W_{\left(1\right)}W_{\left(1\right)}^{T}\right)}^{-\frac{1}{2}}$
4. Updated *W_t_* by $W_{t}={\left(X_{t}^{T}X_{t}+\theta_{1}D+\theta_{2}\bar{\bar{D}}\right)}^{-1}(X_{t}^{T}Y+\theta_{1}D\bar{D})$

**End**

#### Selection of candidate genes with regression coefficients

**Figure 10:**
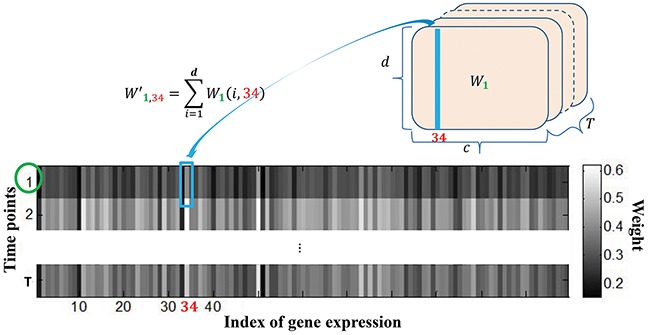
Calculation of the overall weight map of *c* genes with respect to the *d* image features from regression coefficient matrix

Once the regression coefficients were obtained, we identified a compact set of genes whose expression values were highly correlated with the imaging features by two steps. First, we calculated the overall weights for the expression of each gene with respect to all imaging features by the following formulation:

$$W_{t,j}^{'}=\overset{d}{\underset{i=1}{\sum }}W_{t}(i,j),t=1\cdots T,j=1\cdots c$$ (7)

where $W_{t,j}^{'}$ is total weight between the *j*-th gene expression and *d*- mage features at time point *t*, as shown in Figure 10.

We then selected the genes by their average overall weight over the times, i.e., $\frac{1}{T}\sum_{t=1}^{T}W_{t,j}^{'}$. The genes with top-ranked average weight were considered as the candidate genes, which were assumed to be closely related to the imaging features.

## Biological function analysis of the candidate genes

The ultimate goal of our study is to identify the potential genomic biomarkers associated with the PsP and TTP. The potential genomic biomarkers can be used to predict the development of PsP and TTP in the GBM patients after postoperative radiotherapy with concurrent and consolidative TMZ. Thus, candidate biomarkers should be the genes that are significantly differentially expressed in these two groups with roles in tumor suppression or prevention. Once candidate genes were selected by the radiogenomics study, i.e., association using longitudinal sparse regression method, we conducted the biological function analysis to discover the potential genomic biomarkers for PsP and TTP.

First, we performed the functional annotation and pathway enrichment analysis for the candidate genes based on three databases, including Gene Ontology (GO) [56, 57], Human Protein Atlas (HPA) [58, 59], and Ingenuity Pathways Analysis (IPA, Ingenuity System Inc, USA, http://www.ingenuity.com/). The GO Bioinformatics Resource was employed to investigate the enrichment of gene sets. The HPA has been developed to systematically explore the human proteome using Antibody-Based Proteomics. We applied the HPA to check the protein levels of the candidate genes in the GBM tissues. IPA was used to interpret candidate genes in the context of biological processes and canonical pathways. At last, we identified a set of potential biomarkers and summarized the biological mechanisms with existing studies.

## Validation of potential biomarkers using tcga data

To validate the expression level of the identified biomarkers, we collected 6 PsP and 15 TTP samples from TCGA. We employed Wilcoxon rank sum test to calculate the P-value in two groups to test whether the expression levels of identified biomarkers in TCGA were consistent with the dataset from our medical center.

## SUPPLEMENTARY FIGURES AND TABLES








