# Supplementary material for: Identification of biomarkers for pseudo and true progression of GBM based on radiogenomics study
Source: Oncotarget. 2016 Jul 13;7(34):55377–94. doi: 10.18632/oncotarget.10553 (PMC5342424; doi:10.18632/oncotarget.10553)
Supplement: Supplementary file 1 [file oncotarget-07-55377-s001.pdf]

Identification of biomarkers for pseudo and true progression of GBM based on radiogenomics study

SUPPLEMENTARY MATERIALS

Comparison with classic scheme (i.e. directly using the sample labels for marker identification)

We have compared our approach with the classic scheme (i.e. directly using the sample labels for marker identification), as shown in Supplementary Figure. S2 and S3. Actually, our approach also adopted the phenotype labels. Specifically, in the first step, we compared the gene expression levels in the PsP and TTP groups to obtain the differentially expressed genes. We initially identified 119 genes using the Wilcoxon rank sum test with $p<0.005$. After that, we selected 33 candidate genes using associations study between imaging features and gene expression profiles with two advantages: 1) the imaging features were extracted from tumors along the longitudinal MRI and provided diagnostic information of PsP and TTP. Thus, the 33 candidate genes were confirmed to be associated with the development of PsP and TTP. 2) The association study can narrow down candidate genes from 119 to 33, which improves the efficiency of the biological relevance analysis. In summary, the association study based on the differentially expressed genes facilitates the biological analysis in efficiently identifying most relevant signaling pathways.

To illustrate the advantage of our approach, we conducted the comparison experiments (Supplementary Figure. S2 and S3). First, we only used the phenotype labels to obtain 119 differentially expressed genes using the Wilcoxon rank sum test with $p<0.005$. We then performed the biological analysis for the 119 genes without the association study. Top-ranked Canonical Pathways and their genes by IPA were shown in Fig. S3. Obviously, the XRCC1 and its pathways, such as BER pathway, DNA Double-strand Break Repair by Non-Homologous End Joining, and DNA damage-induced 14-3-3σsignaling, in the top-ranked lists of our study (Association study, Supplementary Figure. S2) were absent from the significant lists of the classic scheme (Supplementary Figure S3 (a)). The p-value of these XRCC1 pathways from the classic scheme was slightly greater than 0.05 (Supplementary Figure S3 (b)). Other pathways in the top-ranked list, such as the Role of RIG1-like Receptors in Antiviral innate immunity, the Role of BRCA1 in DAN damage response, and the role of pattern recognition receptors in recognition of bacteria and viruses, were not directly related to cancer development. This result indicates that differentially expressed genes without relation to the PsP and TTP may confound the biological analysis. Therefore, the association study can not only confirm the candidate genes with relation to the development of PsP and TTP but also narrow down the candidate genes, thereby ensuring the real significant genes stand out in the biological analysis.

Effect of morphological features with different size

To illustrate the effect of features, we conducted comparisons of six feature sets with different sizes, as shown in Supplementary Figure S5. The six feature sets were 255 morphological features, 50 clinical morphological features, 50 features randomly selected from our 255 features, and 20 features randomly selected from the 50 clinical features for three times, corresponding to Supplementary Figure S5 (a)-(f), respectively. Specifically, we selected 50 clinical morphological features from 255 features, corresponding to the VASARI, which offers a set of well-defined terms that describe the GBM tumors[1]. The 50 clinical morphological features contain the major/minor axis length of the enhanced region, the thickness of enhancing margin, tumor volume, proportion enhancing and proportion necrosis, etc. In each experiment of the individual feature set, there were 27 parameter combinations for our model. We defined the coverage rate *P* as the occurrence frequency of genes on the lists of weight ranked top 50 in 27 parameter combinations. As a result, we can obtain three different sets of candidate genes with 80%, 90% and 100% of coverage *P* for the individual feature set. Results from three representative feature sets of 255 features, 50 clinical features, and 20 clinical features (corresponding to the Supplementary Figure S5 (a), (b) and (d), respectively) were shown in Supplementary Table S3. Then, the candidate genes identified by coverage $P=0.8$ were used for pathway analysis using IPA. Not surprisingly, the genes from the top-ranked canonical pathways from six feature sets, called pathway genes, were same, as shown in the right column of Supplementary Table S3. Supplementary

Figure S5 shows the gene overlap among candidate genes from different coverage *P* and pathway genes. Obviously, when the feature number is 20 or 50, there is no overlap between candidate genes from *P=100%* and pathway genes. Conversely, our model with 255 features yielded the biomarkers, i.e., IRF9 and XRCC1, in all of the three coverage rates. These results indicate that the performance of our model with 255 features is more robust than that with 50 or 20 features.

REFERENCE

1. Thomas Jefferson University hospital, "VASARI MRI visual feature guide" (2010)

SUPPLEMENTARY FIGURES AND TABLES

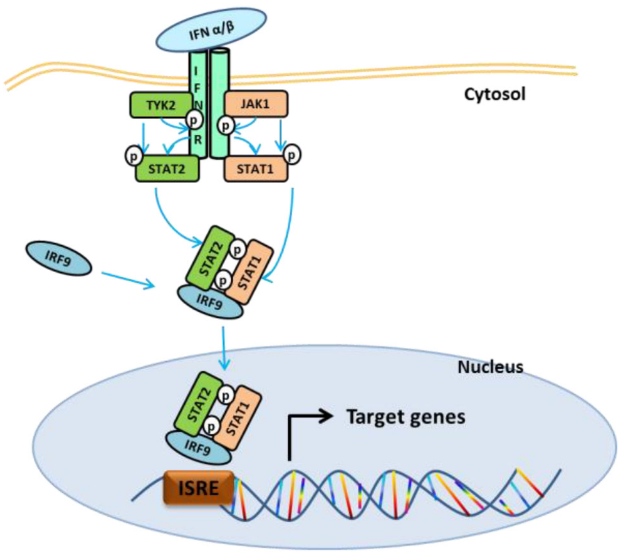

Supplementary Figure S1: JAK-STAT pathway for type I IFN, retrieved and summarized from previous studies.

(a)

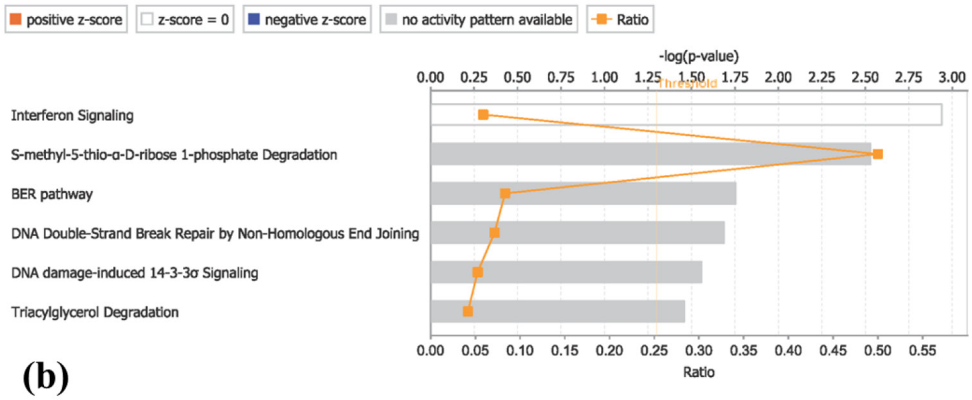

(b)

Ingenuity Canonical Pathways	-log(p-value)	Ratio	Molecules
Interferon Signaling	2.94E00	5.88E-02	IRF9,IFIT3
S-methyl-5-thio-α-D-ribose 1-phosphate Degradation	2.53E00	5E-01	MRI1
BER pathway	1.76E00	8.33E-02	XRCC1
DNA Double-Strand Break Repair by Non-Homologous End Joining	1.69E00	7.14E-02	XRCC1
DNA damage-induced 14-3-3σ Signaling	1.56E00	5.26E-02	RAD9A
Triacylglycerol Degradation	1.46E00	4.17E-02	MGLL

Supplementary Figure S2: Top-ranked canonical pathways associated with the 33 candidate genes selected by radiogenomics. Canonical pathways are ordered by the *p*-values ($p<0.05$).

(a)

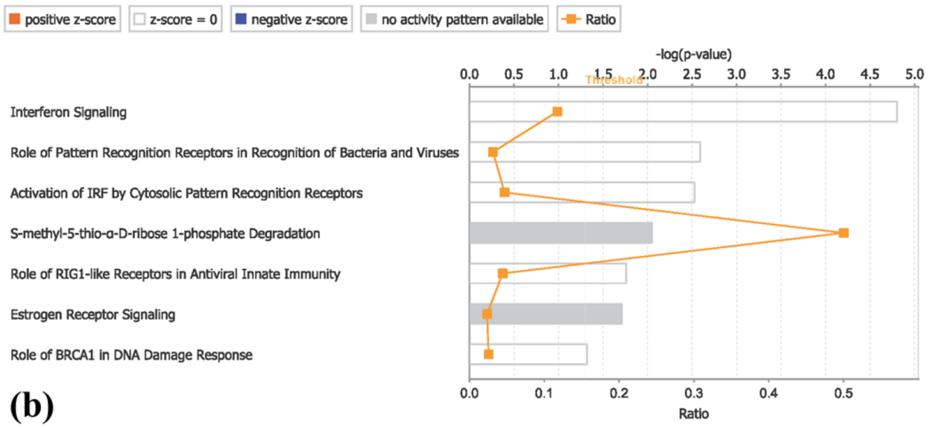

(b)

Ingenuity Canonical Pathways	-log(p-value)	Ratio	Molecules
Interferon Signaling	4.8E00	1.18E-01	OAS1,IRF9,IFIT1,IFIT3
Role of Pattern Recognition Receptors in Recognition of Bacteria and Viruses	2.59E00	3.15E-02	OAS1,OAS3,OAS2,DDX58
Activation of IRF by Cytosolic Pattern Recognition Receptors	2.53E00	4.69E-02	DHX58,IRF9,DDX58
S-methyl-5-thio-α-D-ribose 1-phosphate Degradation	2.05E00	5E-01	MRI1
Role of RIG1-like Receptors in Antiviral Innate Immunity	1.76E00	4.44E-02	DHX58,DDX58
Estrogen Receptor Signaling	1.71E00	2.36E-02	MED31,MED12,TAF1
Role of BRCA1 in DNA Damage Response	1.32E00	2.56E-02	FAM175A,ATF1
BER pathway	1.28E00	8.33E-02	XRCC1
DNA Double-Strand Break Repair by Non-Homologous End Joining	1.22E00	7.14E-02	XRCC1
DNA damage-induced 14-3-3σ Signaling	1.09E00	5.26E-02	RAD9A
Triacylglycerol Degradation	9.91E-01	4.17E-02	MGLL

Supplementary Figure S3: Top-ranked canonical pathways associated with the 119 differentially expressed genes. Top-ranked canonical pathways were highlighted red in (b) with $p<0.05$.

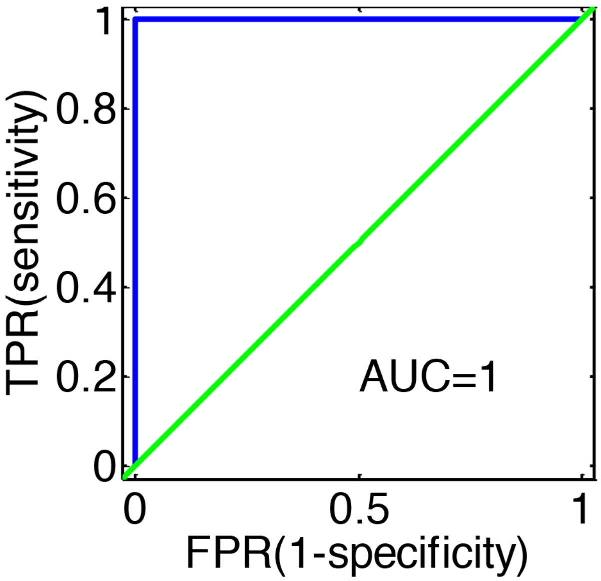

Supplementary Figure S4: The ROC curve for the classification of the 119 differentially expressed genes as features. The SVM classifier and fivefold cross-validation were applied in this study. *sensitivity=TP/(TP+FN)* and *specificity=TN/(FP+TN)*, where *TP*, *FP* , *FN*, and *TN* refer to true positive, false positive, false negative, and true negative, respectively.

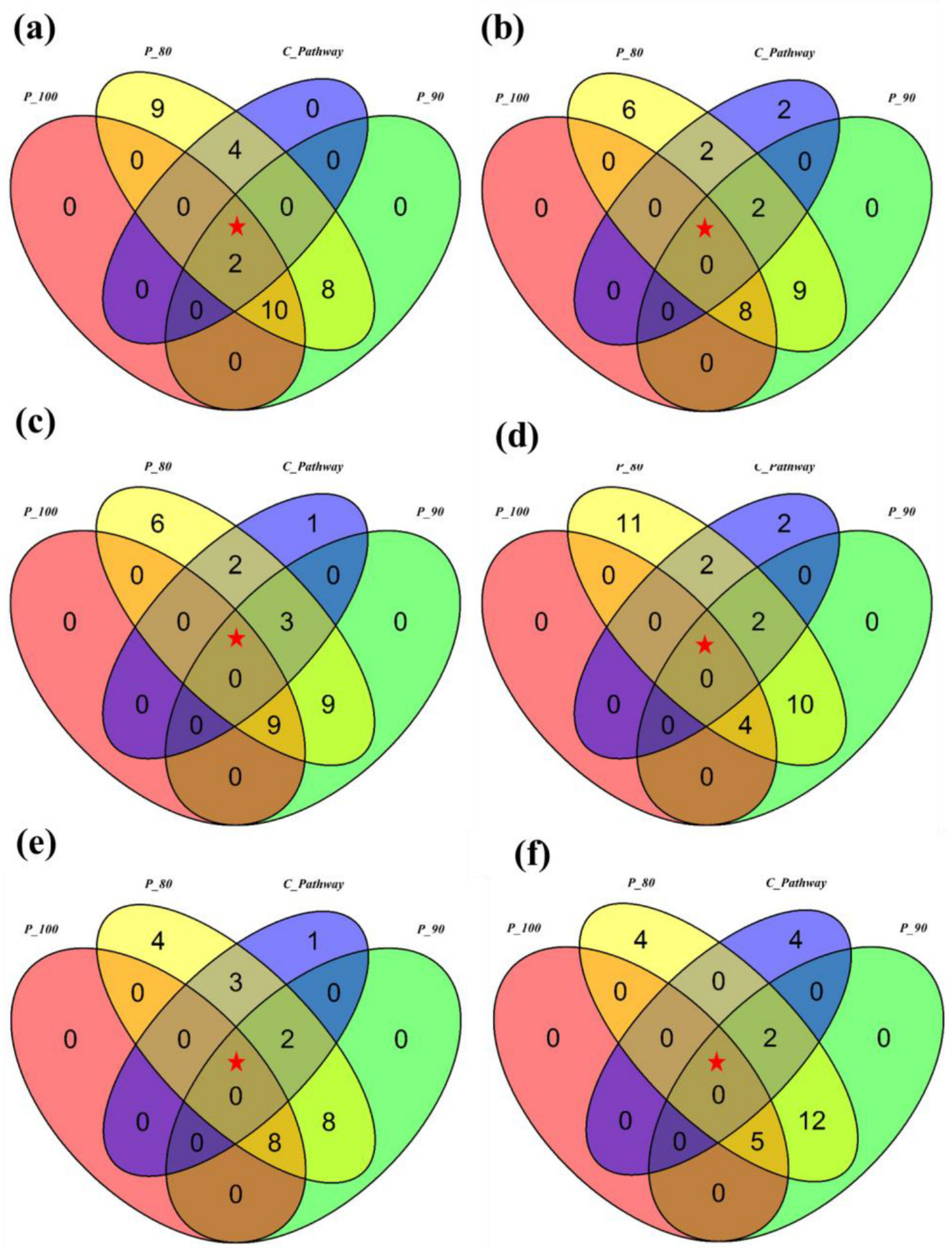

Supplementary Figure S5: The gene overlaps among candidate genes from different coverage rates *P* and genes from top-ranked canonical pathways (i.e., *C_Pathway*). The coverage rate *P* was defined as the occurrence frequency of genes on the lists of weight ranker top 50 in 27 parameter combinations. *P_100, P_90 and P_80* mean 100%, 90% and 80% of coverage *P*, respectively. Experiments with six feature sets: **a.** 225 image features; **b.** 50 image features used in clinic practices; **c.** 50 image features randomly selected from **a.**; **d.-f.** 20 image features randomly selected from the 50 clinical features in **b.** for three times.

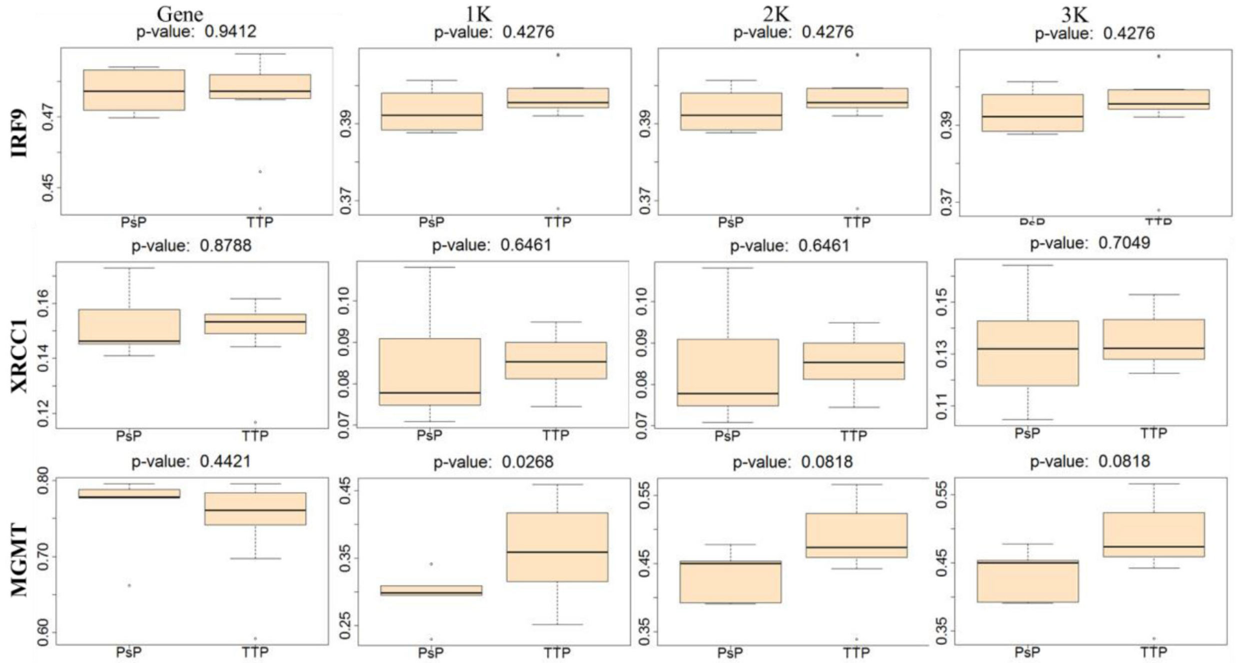

Supplementary Figure S6: Boxplots of gene methylation, promoter methylation with 1k, 2k, and 3k window of TSS from 1 to 4 columns, respectively, for IRF9 (1st row), XRCC1(2nd row), and MGMT (3rd row).

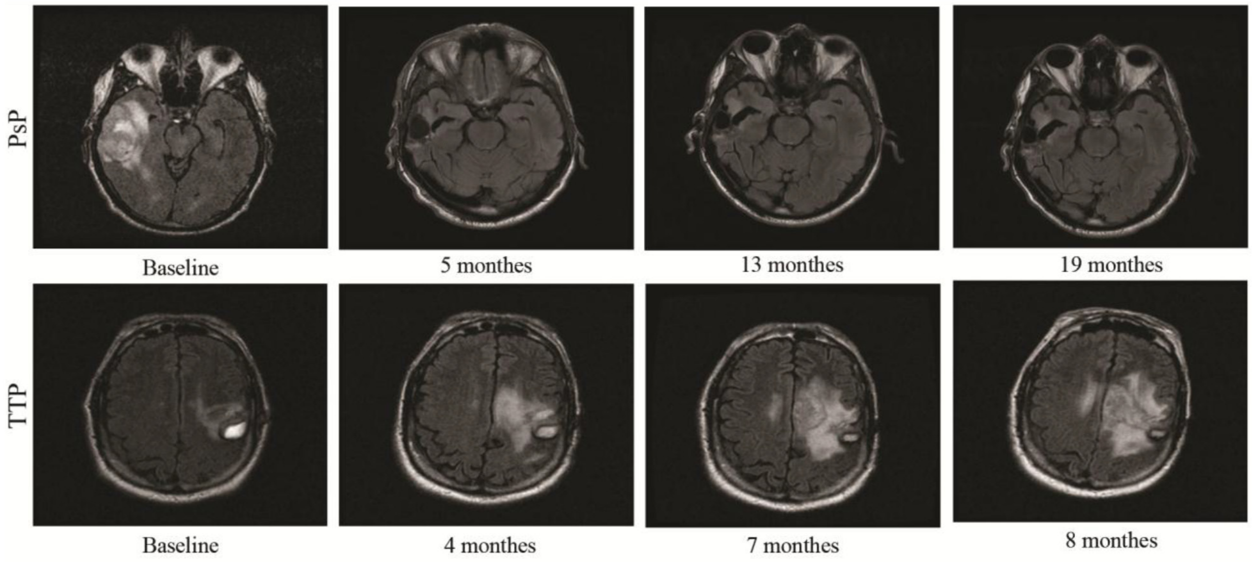

Supplementary Figure S7: Demonstration of PsP and TTP based on longitudinal MRI. All of the images are from TCIA. The sample ID for the top row images is TCGA-06-0185, and the bottom row is TCGA-06-1084.

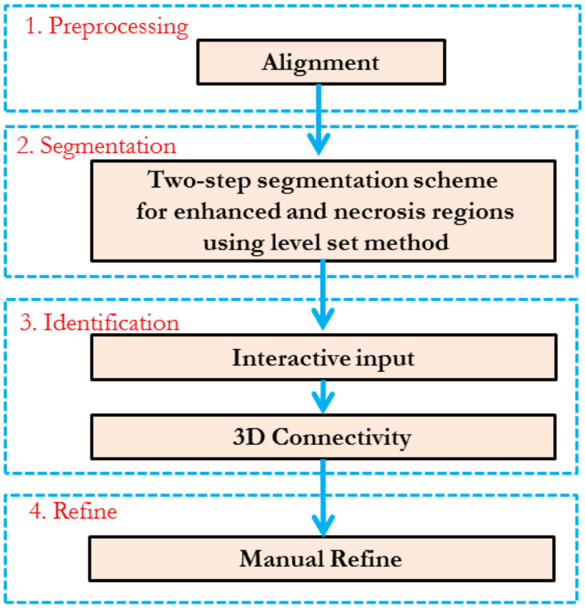

Supplementary Figure S8: The flowchart of the segmentation of tumor regions (i.e., enhanced region and necrosis regions).

Supplementary Table S1: Summary of all the features extracted from the longitudinal MRI.

See Supplementary File 1

Supplementary Table S2: Top 119 significant genes screened by the wilcoxon rank sum test with P-value of 0.005.

See Supplementary File 2

Supplementary Table S3: The candidate genes selected from three representative feature sets of 255 features, 50 clinical features and 20 clinical features (corresponding to the Figure R1 (a), (b) and (d), respectively). *P_100, P_90 and P_80* mean 100%, 90% and 80% of coverage *P*, respectively. The genes from top ranked canonical pathways were in the right column.

See Supplementary File 3

Supplementary Table S4: Predication of MGMT methylation status in previous studies

		Methylated MGMT		Unmethylated MGMT		Sensitivity (1)	Specificity
Brandes, et al[1]	PsP	21	91.3%	11	40.7%	65.6%	88.9%
	TTP	2	8.7%	16	59.3%		
Motegi, et al[2]	PsP	2	40%	1	16.7%	66.7%	62.5%
	TTP	3	60%	5	83.3%		
Kong, et al[3]	PsP	15	37.5%	8	24.2%	65.1%	50%
	TTP	25	62.5%	25	75.8%		
Mieghem, et al[4] (2)	PsP	9	37.5%	4	12.9%	69.2%	64.3%
	TTP	15	62.5%	27	87.1%		
Park, et al[5] (3)	PsP	4	80%	7	35%	36.4%	92.9%
	TTP	1	20%	13	65%		

(1): Sensitivity and specificity were defined for detecting the PsP based on methylated MGMT; (2): MGMT assessed using liberal criteria; (3) MGMT promoter methylation status determined by methylation-specific polymerase chain reaction.

Reference:

[1] A.A. Brandes, E. Franceschi, A. Tosoni, V. Blatt, A. Pession, G. Tallini, R. Bertorelle, S. Bartolini, F. Calbucci, A. Andreoli, G. Frezza, M. Leonardi, F. Spagnolli, M. Ermani, “MGMT promoter methylation status can predict the incidence and outcome of pseudoprogression after concomitant radiochemotherapy in newly diagnosed glioblastoma patients,” Journal of Clinical Oncology 26, 2192-2197 (2008).

[2] H. Motegi, Y. Kamoshima, S. Terasaka, H. Kobayashi, S. Yamaguchi, M. Tanino, J. Murata, K. Houkin, “IDH1 mutation as a potential novel biomarker for distinguishing pseudoprogression from true progression in patients with glioblastoma treated with temozolomide and radiotherapy,” Brain Tumor Pathol 30, 67-72 (2013).

[3] D.S. Kong, S.T. Kim, E.H. Kim, D.H. Lim, W.S. Kim, Y.L. Suh, J.I. Lee, K. Park, J.H. Kim, D.H. Nam, “Diagnostic dilemma of pseudoprogression in the treatment of newly diagnosed glioblastomas: the role of assessing relative cerebral blood flow volume and oxygen-6-methylguanine-DNA methyltransferase promoter methylation status,” AJNR Am J Neuroradiol 32, 382-387 (2011).

[4] E. Van Mieghem, A. Wozniak, Y. Geussens, J. Menten, S. De Vleeschouwer, F. Van Calenbergh, R. Sciot, S. Van Gool, O.E. Bechter, P. Demaerel, G. Wilms, P.M. Clement, “Defining pseudoprogression in glioblastoma multiforme,” Eur J Neurol 20, 1335-1341 (2013).

[5] C.K. Park, J. Kim, S.Y. Yim, A.R. Lee, J.H. Han, C.Y. Kim, S.H. Park, T.M. Kim, S.H. Lee, S.H. Choi, S.K. Kim, D.G. Kim, H.W. Jung, “Usefulness of MS-MLPA for detection of MGMT promoter methylation in the evaluation of pseudoprogression in glioblastoma patients,” Neuro Oncol 13, 195-202 (2011).

Supplementary Table S5: Gene expressions of IRF9 and XRCC1 in the samples from TCGA

Patient ID	Type (PsP or TTP)	IRF9	XRCC1
TCGA-06-0125	PsP	8.156670711	6.920976104
TCGA-06-0132	TTP	8.478463523	4.926175355
TCGA-06-0138	TTP	7.747738065	5.101606898
TCGA-06-0143	TTP	8.086957245	5.626329777
TCGA-06-0156	TTP	7.578428437	5.655630949
TCGA-06-0166	TTP	7.934104041	5.746312365
TCGA-06-0171	TTP	8.400817171	5.520019494
TCGA-06-0185	PsP	8.894900451	6.044437145
TCGA-06-0188	TTP	8.500423967	5.577020075
TCGA-06-0881	PsP	9.189752485	5.954228976
TCGA-06-1084	TTP	7.645471537	5.196564308
TCGA-14-0783	TTP	8.713694229	5.45244386
TCGA-14-0865	TTP	8.288237374	5.845771704
TCGA-14-1037	PsP	9.226101835	6.326896707
TCGA-14-1402	TTP	8.115959165	5.700478983
TCGA-14-1454	TTP	7.776848448	6.146345705
TCGA-14-1456	PsP	8.399362815	6.416399775
TCGA-14-1459	TTP	7.381387031	5.539365678
TCGA-14-1821	PsP	9.321442885	6.562523335
TCGA-14-1823	TTP	8.29498316	5.399658914
TCGA-14-1829	TTP	7.277918194	5.106995763

The sample type, i.e., pseudoprogression (PsP) or true tumor progression (TTP), is determined by the longitudinal MRI from TCIA.
