# Supplementary material for: Identification of biomarkers for pseudo and true progression of GBM based on radiogenomics study
Source: Oncotarget. 2016 Jul 13;7(34):55377–94. doi: 10.18632/oncotarget.10553 (PMC5342424; doi:10.18632/oncotarget.10553)
Supplement: Supplementary file 2 [file oncotarget-07-55377-s002.docx]

| **Features extraction from the enhanced region (1-80: total sum, representative case, mean, max and min value)** |
| --- |
| ***Total sum of features from slices in a case 1-16***  ***1*** Area ration of enhanced region and necrosis region  ***2*** Area of enhanced region  ***3*** Length of enhanced region  ***4*** Width of enhanced region  ***5*** Radius of the enhanced region considered as circle region  ***6*** Regions number in the enhanced region  ***7*** Maximum area in the enhanced regions  ***8*** Perimeter of the enhanced region with maximum area  ***9*** Eccentricity of the enhanced region with maximum area  ***10*** Compactness of the enhanced region with maximum area  ***11*** Sphericity of the enhanced region with maximum area  ***12*** Orientation of the enhanced region with maximum area  ***13*** Bounding box area of the enhanced region with maximum area  ***14*** Major axis length of the enhanced region with maximum area  ***15*** Minor axis length of the enhanced region with maximum area  ***16*** Solidity of the enhanced region with maximum area  ***Feature from the slice with biggest tumor area in a case 17-32***  ***17*** Area ration of enhanced region and necrosis region  ***18*** Area of enhanced region  ***19*** Length of enhanced region  ***20*** Width of enhanced region  ***21*** Radius of the enhanced region considered as circle region  ***22*** Regions number in the enhanced region  ***23*** Maximum area in the enhanced regions  ***24*** Perimeter of the enhanced region with maximum area  ***25*** Eccentricity of the enhanced region with maximum area  ***26*** Compactness of the enhanced region with maximum area  ***27*** Sphericity of the enhanced region with maximum area  ***28*** Orientation of the enhanced region with maximum area  ***29*** Bounding box area of the enhanced region with maximum area  ***30*** Major axis length of the enhanced region with maximum area  ***31*** Minor axis length of the enhanced region with maximum area  ***32*** Solidity of the enhanced region with maximum area  ***Mean value of features extracted from a case 33-48***  ***33*** Area ration of enhanced region and necrosis region  ***34*** Area of enhanced region  ***35*** Length of enhanced region  ***36*** Width of enhanced region  ***37*** Radius of the enhanced region considered as circle region  ***38*** Regions number in the enhanced region  ***39*** Maximum area in the enhanced regions  ***40*** Perimeter of the enhanced region with maximum area  ***41*** Eccentricity of the enhanced region with maximum area  ***42*** Compactness of the enhanced region with maximum area  ***43*** Sphericity of the enhanced region with maximum area  ***44*** Orientation of the enhanced region with maximum area  ***45*** Bounding box area of the enhanced region with maximum area  ***46*** Major axis length of the enhanced region with maximum area  ***47*** Minor axis length of the enhanced region with maximum area  ***48*** Solidity of the enhanced region with maximum area  ***Maximum value of features extracted from a case 49-64***  ***49*** Area ration of enhanced region and necrosis region  ***50*** Area of enhanced region  ***51*** Length of enhanced region  ***52*** Width of enhanced region  ***53*** Radius of the enhanced region considered as circle region  ***54*** Regions number in the enhanced region  ***55*** Maximum area in the enhanced regions  ***56*** Perimeter of the enhanced region with maximum area  ***57*** Eccentricity of the enhanced region with maximum area  ***58*** Compactness of the enhanced region with maximum area  ***59*** Sphericity of the enhanced region with maximum area  ***60*** Orientation of the enhanced region with maximum area  ***61*** Bounding box area of the enhanced region with maximum area  ***62*** Major axis length of the enhanced region with maximum area  ***63*** Minor axis length of the enhanced region with maximum area  ***64*** Solidity of the enhanced region with maximum area  ***Minimum value of features extracted from a case 65-80***  ***65*** Area ration of enhanced region and necrosis region  ***66*** Area of enhanced region  ***67*** Length of enhanced region  ***68*** Width of enhanced region  ***69*** Radius of the enhanced region considered as circle region  ***70*** Regions number in the enhanced region  ***71*** Maximum area in the enhanced regions  ***72*** Perimeter of the enhanced region with maximum area  ***73*** Eccentricity of the enhanced region with maximum area  ***74*** Compactness of the enhanced region with maximum area  ***75*** Sphericity of the enhanced region with maximum area  ***76*** Orientation of the enhanced region with maximum area  ***77*** Bounding box area of the enhanced region with maximum area  ***78*** Major axis length of the enhanced region with maximum area  ***79*** Minor axis length of the enhanced region with maximum area  ***80*** Solidity of the enhanced region with maximum area |
| **Features extraction from the necrosis region (81-150: total sum, representative case, mean, max and min value)** |
| ***Total sum of features from slices in a case 81-94***  ***81*** Area ration of enhanced region and necrosis region  ***82*** Area of necrosis region  ***83*** Radius of the necrosis region considered as circle region  ***84*** Regions number in the necrosis region  ***85*** Maximum area in the necrosis regions  ***86*** Perimeter of the necrosis region with maximum area  ***87*** Eccentricity of the necrosis region with maximum area  ***88*** Compactness of the necrosis region with maximum area  ***89*** Sphericity of the necrosis region with maximum area  ***90*** Orientation of the necrosis region with maximum area  ***91*** Bounding box area of the necrosis region with maximum area  ***92*** Major axis length of the necrosis region with maximum area  ***93*** Minor axis length of the necrosis region with maximum area  ***94*** Solidity of the necrosis region with maximum area  ***Feature from the slice with biggest tumor area in a case 95-108***  ***95*** Area ration of enhanced region and necrosis region  ***96*** Area of necrosis region  ***97*** Radius of the necrosis region considered as circle region  ***98*** Regions number in the necrosis region  ***99*** Maximum area in the necrosis regions  ***100*** Perimeter of the necrosis region with maximum area  ***101*** Eccentricity of the necrosis region with maximum area  ***102*** Compactness of the necrosis region with maximum area  ***103*** Sphericity of the necrosis region with maximum area  ***104*** Orientation of the necrosis region with maximum area  ***105*** Bounding box area of the necrosis region with maximum area  ***106*** Major axis length of the necrosis region with maximum area  ***107*** Minor axis length of the necrosis region with maximum area  ***108*** Solidity of the necrosis region with maximum area  ***Mean value of features extracted from a case 109-122***  ***109*** Area ration of enhanced region and necrosis region  ***110*** Area of necrosis region  ***111*** Radius of the necrosis region considered as circle region  ***112*** Regions number in the necrosis region  ***113*** Maximum area in the necrosis regions  ***114*** Perimeter of the necrosis region with maximum area  ***115*** Eccentricity of the necrosis region with maximum area  ***116*** Compactness of the necrosis region with maximum area  ***117*** Sphericity of the necrosis region with maximum area  ***118*** Orientation of the necrosis region with maximum area  ***119*** Bounding box area of the necrosis region with maximum area  ***120*** Major axis length of the necrosis region with maximum area  ***121*** Minor axis length of the necrosis region with maximum area  ***122*** Solidity of the necrosis region with maximum area  ***Maximum value of features extracted from a case 123-136***  ***123*** Area ration of enhanced region and necrosis region  ***124*** Area of necrosis region  ***125*** Radius of the necrosis region considered as circle region  ***126*** Regions number in the necrosis region  ***127*** Maximum area in the necrosis regions  ***128*** Perimeter of the necrosis region with maximum area  ***129*** Eccentricity of the necrosis region with maximum area  ***130*** Compactness of the necrosis region with maximum area  ***131*** Sphericity of the necrosis region with maximum area  ***132*** Orientation of the necrosis region with maximum area  ***133*** Bounding box area of the necrosis region with maximum area  ***134*** Major axis length of the necrosis region with maximum area  ***135*** Minor axis length of the necrosis region with maximum area  ***136*** Solidity of the necrosis region with maximum area  ***Minimum value of features extracted from a case 137-150***  ***137*** Area ration of enhanced region and necrosis region  ***138*** Area of necrosis region  ***139*** Radius of the necrosis region considered as circle region  ***140*** Regions number in the necrosis region  ***141*** Maximum area in the necrosis regions  ***142*** Perimeter of the necrosis region with maximum area  ***143*** Eccentricity of the necrosis region with maximum area  ***144*** Compactness of the necrosis region with maximum area  ***145*** Sphericity of the necrosis region with maximum area  ***146*** Orientation of the necrosis region with maximum area  ***147*** Bounding box area of the necrosis region with maximum area  ***148*** Major axis length of the necrosis region with maximum area  ***149*** Minor axis length of the necrosis region with maximum area  ***150*** Solidity of the necrosis region with maximum area |
| **Features extraction from the tumor region (151-225: total sum, representative case, mean, max and min value)** |
| ***Total sum of features from slices in a case 151-165***  ***151*** Area of tumor region  ***152*** x coordinate of centroid point  ***153*** y coordinate of centroid point  ***154***  Radius of the tumor region considered as circle region  ***155*** Regions number in the tumor region  ***156*** Maximum area in the tumor regions  ***157*** Perimeter of the tumor region with maximum area  ***158*** Eccentricity of the tumor region with maximum area  ***159*** Compactness of the tumor region with maximum area  ***160*** Sphericity of the tumor region with maximum area  ***161*** Orientation of the tumor region with maximum area  ***162*** Bounding box area of the tumor region with maximum area  ***163*** Major axis length of the tumor region with maximum area  ***164*** Minor axis length of the tumor region with maximum area  ***165*** Solidity of the tumor region with maximum area  ***Feature from the slice with biggest tumor area in a case 166-180***  ***166*** Area of tumor region  ***167*** x coordinate of centroid point  ***168*** y coordinate of centroid point  ***169***  Radius of the tumor region considered as circle region  ***170*** Regions number in the tumor region  ***171*** Maximum area in the tumor regions  ***172*** Perimeter of the tumor region with maximum area  ***173*** Eccentricity of the tumor region with maximum area  ***174*** Compactness of the tumor region with maximum area  ***175*** Sphericity of the tumor region with maximum area  ***176*** Orientation of the tumor region with maximum area  ***177*** Bounding box area of the tumor region with maximum area  ***178*** Major axis length of the tumor region with maximum area  ***179*** Minor axis length of the tumor region with maximum area  ***180*** Solidity of the tumor region with maximum area  ***Mean value of features extracted from a case 181-195***  ***181*** Area of tumor region  ***182*** x coordinate of centroid point  ***183*** y coordinate of centroid point  ***184***  Radius of the tumor region considered as circle region  ***185*** Regions number in the tumor region  ***186*** Maximum area in the tumor regions  ***187*** Perimeter of the tumor region with maximum area  ***188*** Eccentricity of the tumor region with maximum area  ***189*** Compactness of the tumor region with maximum area  ***190*** Sphericity of the tumor region with maximum area  ***191*** Orientation of the tumor region with maximum area  ***192*** Bounding box area of the tumor region with maximum area  ***193*** Major axis length of the tumor region with maximum area  ***194*** Minor axis length of the tumor region with maximum area  ***195*** Solidity of the tumor region with maximum area  ***Maximum value of features extracted from a case 196-210***  ***196*** Area of tumor region  ***197*** x coordinate of centroid point  ***198*** y coordinate of centroid point  ***199***  Radius of the tumor region considered as circle region  ***200*** Regions number in the tumor region  ***201*** Maximum area in the tumor regions  ***202*** Perimeter of the tumor region with maximum area  ***203*** Eccentricity of the tumor region with maximum area  ***204*** Compactness of the tumor region with maximum area  ***205*** Sphericity of the tumor region with maximum area  ***206*** Orientation of the tumor region with maximum area  ***207*** Bounding box area of the tumor region with maximum area  ***208*** Major axis length of the tumor region with maximum area  ***209*** Minor axis length of the tumor region with maximum area  ***210*** Solidity of the tumor region with maximum area  ***Maximum value of features extracted from a case 211-225***  ***211*** Area of tumor region  ***212*** x coordinate of centroid point  ***213*** y coordinate of centroid point  ***214***  Radius of the tumor region considered as circle region  ***215*** Regions number in the tumor region  ***216*** Maximum area in the tumor regions  ***217*** Perimeter of the tumor region with maximum area  ***218*** Eccentricity of the tumor region with maximum area  ***219*** Compactness of the tumor region with maximum area  ***220*** Sphericity of the tumor region with maximum area  ***221*** Orientation of the tumor region with maximum area  ***222*** Bounding box area of the tumor region with maximum area  ***223*** Major axis length of the tumor region with maximum area  ***224*** Minor axis length of the tumor region with maximum area  ***225*** Solidity of the tumor region with maximum area |
