# Supplementary material for: Identification of biomarkers for pseudo and true progression of GBM based on radiogenomics study
Source: Oncotarget. 2016 Jul 13;7(34):55377–94. doi: 10.18632/oncotarget.10553 (PMC5342424; doi:10.18632/oncotarget.10553)
Supplement: Supplementary file 3 [file oncotarget-07-55377-s003.docx]

| Rand | SYMBOL | SEQID | P-Value |
| --- | --- | --- | --- |
| 1 | --- | --- | 0.000323 |
| 2 | AP1M1 | NM_001130524 | 0.000323 |
| 3 | ARMC9 | ENST00000359743 | 0.000323 |
| 4 | ATF1 | NM_005171 | 0.000323 |
| 5 | ATP7A | NM_000052 | 0.000323 |
| 6 | C19orf66 | ENST00000253110 | 0.000323 |
| 7 | CAMK2N2 | NM_033259 | 0.000323 |
| 8 | FAM123B | NM_152424 | 0.000323 |
| 9 | HERC5 | NM_016323 | 0.000323 |
| 10 | HIST1H4G | NM_003547 | 0.000323 |
| 11 | IFI44L | NM_006820 | 0.000323 |
| 12 | IFIT3 | NM_001031683 | 0.000323 |
| 13 | IRF9 | NM_006084 | 0.000323 |
| 14 | NTNG1 | NM_001113226 | 0.000323 |
| 15 | OAS3 | ENST00000228928 | 0.000323 |
| 16 | TULP3 | NM_003324 | 0.000323 |
| 17 | USP18 | ENST00000215794 | 0.000323 |
| 18 | --- | --- | 0.000646 |
| 19 | --- | --- | 0.000646 |
| 20 | C1GALT1C1 | NM_152692 | 0.000646 |
| 21 | DHX58 | NM_024119 | 0.000646 |
| 22 | DMRTA1 | NM_022160 | 0.000646 |
| 23 | DYNLL2 | NM_080677 | 0.000646 |
| 24 | HERC6 | NM_017912 | 0.000646 |
| 25 | TAS2R19 | NM_176888 | 0.000646 |
| 26 | --- | --- | 0.001293 |
| 27 | --- | --- | 0.001293 |
| 28 | --- | --- | 0.001293 |
| 29 | APBB3 | ENST00000357560 | 0.001293 |
| 30 | CCDC77 | NM_032358 | 0.001293 |
| 31 | DDX60 | NM_017631 | 0.001293 |
| 32 | FAM122C | NM_001170779 | 0.001293 |
| 33 | FBXO40 | ENST00000338040 | 0.001293 |
| 34 | IFI6 | NM_002038 | 0.001293 |
| 35 | IGF1R | NM_000875 | 0.001293 |
| 36 | MDM1 | NM_017440 | 0.001293 |
| 37 | MED12 | NM_005120 | 0.001293 |
| 38 | MGLL | ENST00000434178 | 0.001293 |
| 39 | MTFMT | NM_139242 | 0.001293 |
| 40 | OR10A2 | ENST00000307322 | 0.001293 |
| 41 | PARP12 | ENST00000263549 | 0.001293 |
| 42 | PHF11 | NM_001040443 | 0.001293 |
| 43 | RASGEF1C | ENST00000393371 | 0.001293 |
| 44 | U2AF1L4 | NM_001040425 | 0.001293 |
| 45 | ZNF214 | NM_013249 | 0.001293 |
| 46 | ZNF529 | NM_020951 | 0.001293 |
| 47 | ZNF563 | NM_145276 | 0.001293 |
| 48 | --- | --- | 0.001293 |
| 49 | --- | --- | 0.002262 |
| 50 | --- | --- | 0.002262 |
| 51 | --- | --- | 0.002262 |
| 52 | BAZ2A | NM_013449 | 0.002262 |
| 53 | CASQ1 | NM_001231 | 0.002262 |
| 54 | CCDC41 | NM_016122 | 0.002262 |
| 55 | DACT1 | NM_016651 | 0.002262 |
| 56 | DDX58 | NM_014314 | 0.002262 |
| 57 | ERCC1 | NM_001983 | 0.002262 |
| 58 | FLJ33360 | NR_028351 | 0.002262 |
| 59 | IFI44 | NM_006417 | 0.002262 |
| 60 | OAS1 | ENST00000202917 | 0.002262 |
| 61 | OAS2 | ENST00000392583 | 0.002262 |
| 62 | PHF8 | NM_015107 | 0.002262 |
| 63 | RAD9A | NM_004584 | 0.002262 |
| 64 | TAF1 | NM_004606 | 0.002262 |
| 65 | TIAM2 | NM_001010927 | 0.002262 |
| 66 | USP28 | ENST00000003302 | 0.002262 |
| 67 | XRCC1 | ENST00000458471 | 0.002262 |
| 68 | ZFP28 | NM_020828 | 0.002262 |
| 69 | ZNF256 | NM_005773 | 0.002262 |
| 70 | ZNF266 | NM_006631 | 0.002262 |
| 71 | --- | --- | 0.002262 |
| 72 | --- | --- | 0.002262 |
| 73 | --- | --- | 0.003878 |
| 74 | --- | --- | 0.003878 |
| 75 | --- | --- | 0.003878 |
| 76 | --- | --- | 0.003878 |
| 77 | --- | --- | 0.003878 |
| 78 | --- | --- | 0.003878 |
| 79 | ATF7 | NM_006856 | 0.003878 |
| 80 | C17orf65 | NR_049729 | 0.003878 |
| 81 | C2CD3 | NM_015531 | 0.003878 |
| 82 | DNAJC9 | ENST00000372950 | 0.003878 |
| 83 | DYX1C1 | NM_130810 | 0.003878 |
| 84 | EPHB2 | NM_017449 | 0.003878 |
| 85 | FAM175A | ENST00000321945 | 0.003878 |
| 86 | FHL5 | NM_020482 | 0.003878 |
| 87 | FMR1 | NM_002024 | 0.003878 |
| 88 | FOXJ2 | ENST00000162391 | 0.003878 |
| 89 | GRK4 | NM_182982 | 0.003878 |
| 90 | IFIT1 | NM_001548 | 0.003878 |
| 91 | KDM5A | NM_001042603 | 0.003878 |
| 92 | KIAA0753 | ENST00000361413 | 0.003878 |
| 93 | LINC00310 | NR_027267 | 0.003878 |
| 94 | LOC100132686 | BC020894 | 0.003878 |
| 95 | LRRTM1 | ENST00000433224 | 0.003878 |
| 96 | MED31 | NM_016060 | 0.003878 |
| 97 | MRI1 | NM_001031727 | 0.003878 |
| 98 | MRPS6 | NM_032476 | 0.003878 |
| 99 | MTCP1NB | ENST00000369479 | 0.003878 |
| 100 | NAF1 | NM_138386 | 0.003878 |
| 101 | OR52H1 | ENST00000322653 | 0.003878 |
| 102 | PCDHB13 | NM_018933 | 0.003878 |
| 103 | PHKA2 | ENST00000379942 | 0.003878 |
| 104 | PKD1L1 | ENST00000462350 | 0.003878 |
| 105 | RAB8A | NM_005370 | 0.003878 |
| 106 | RIBC1 | NM_001267053 | 0.003878 |
| 107 | RSAD1 | NM_018346 | 0.003878 |
| 108 | SLC25A14 | NM_003951 | 0.003878 |
| 109 | TAB3 | NM_152787 | 0.003878 |
| 110 | TCTN2 | NM_024809 | 0.003878 |
| 111 | TINAGL1 | NM_022164 | 0.003878 |
| 112 | TRDMT1 | NM_004412 | 0.003878 |
| 113 | TRIM21 | NM_003141 | 0.003878 |
| 114 | TTC23 | NM_001040655 | 0.003878 |
| 115 | UBQLNL | NM_145053 | 0.003878 |
| 116 | UNC119B | NM_001080533 | 0.003878 |
| 117 | ZNF471 | NM_020813 | 0.003878 |
| 118 | ZNF550 | ENST00000325134 | 0.003878 |
| 119 | ZNF701 | ENST00000301093 | 0.003878 |
