# Supplementary material for: Identification of biomarkers for pseudo and true progression of GBM based on radiogenomics study
Source: Oncotarget. 2016 Jul 13;7(34):55377–94. doi: 10.18632/oncotarget.10553 (PMC5342424; doi:10.18632/oncotarget.10553)
Supplement: Supplementary file 4 [file oncotarget-07-55377-s004.docx]

| **255 features (a)** | | | **50 clinical features (b)** | | | **20 clinical features (d)** | | | **Pathway** |
| --- | --- | --- | --- | --- | --- | --- | --- | --- | --- |
| **P_80** | **P_90** | **P_100** | **P_80** | **P_90** | **P_100** | **P_80** | **P_90** | **P_100** |  |
| AP1M1  C19orf66  IFI44L  IFIT3  IRF9  OAS3  TULP3  USP18  DYNLL2  TAS2R19  APBB3  FAM122C  IFI6  MED12  MGLL  BAZ2A  ERCC1  PHF8  RAD9A  XRCC1  ATF7  C17orf65  C2CD3  DNAJC9  FMR1  FOXJ2  KDM5A  KIAA0753  MRI1  MRPS6  RAB8A  TAB3  TCTN2 | AP1M1  C19orf66  IFI44L  IRF9  TULP3  DYNLL2  APBB3  IFI6  MED12  BAZ2A  ERCC1  PHF8  XRCC1  ATF7  C2CD3  DNAJC9  MRPS6  RAB8A  TAB3  TCTN2 | AP1M1  C19orf66  IFI44L  IRF9  TULP3  DYNLL2  APBB3  IFI6  XRCC1  ATF7  DNAJC9  MRPS6 | AP1M1  C19orf66  IFI44L  IRF9  OAS3  TULP3  DYNLL2  TAS2R19  APBB3  IFI6  MED12  MGLL  BAZ2A  ERCC1  OAS1  PHF8  RAD9A  XRCC1  ATF7  C17orf65  FMR1  FOXJ2  KDM5A  MRPS6  RAB8A  TAB3  TCTN2 | AP1M1  C19orf66  IFI44L  IRF9  TULP3  DYNLL2  APBB3  IFI6  MED12  BAZ2A  ERCC1  PHF8  XRCC1  ATF7  FMR1  FOXJ2  MRPS6  TAB3  TCTN2 | C19orf66  IFI44L  TULP3  APBB3  IFI6  MED12  BAZ2A  MRPS6 | AP1M1  C19orf66  IFI44L  IRF9  OAS3  TULP3  USP18  DYNLL2  TAS2R19  APBB3  FAM122C  IFI6  MED12  BAZ2A  ERCC1  OAS1  PHF8  RAD9A  XRCC1  ATF7  DNAJC9  FMR1  FOXJ2  KDM5A  MRI1  MRPS6  RAB8A  TAB3  TCTN2 | AP1M1  C19orf66  IFI44L  IRF9  TULP3  DYNLL2  APBB3  IFI6  MED12  BAZ2A  ERCC1  PHF8  XRCC1  ATF7  FOXJ2  MRPS6 | IFI44L  IFI6  BAZ2A  ATF7 | IRF9  XRCC1  IFIT3  MRI1  RAD9A  MGLL |
